# Novel Aza-Paternò-Büchi Reaction Allows Pinpointing Carbon–Carbon Double Bonds in Unsaturated Lipids by Higher Collisional Dissociation

**DOI:** 10.1021/acs.analchem.2c02549

**Published:** 2022-09-19

**Authors:** Andrea Cerrato, Anna Laura Capriotti, Chiara Cavaliere, Carmela Maria Montone, Susy Piovesana, Aldo Laganà

**Affiliations:** Department of Chemistry, Sapienza University of Rome, Piazzale Aldo Moro 5, Rome 00185, Italy

## Abstract

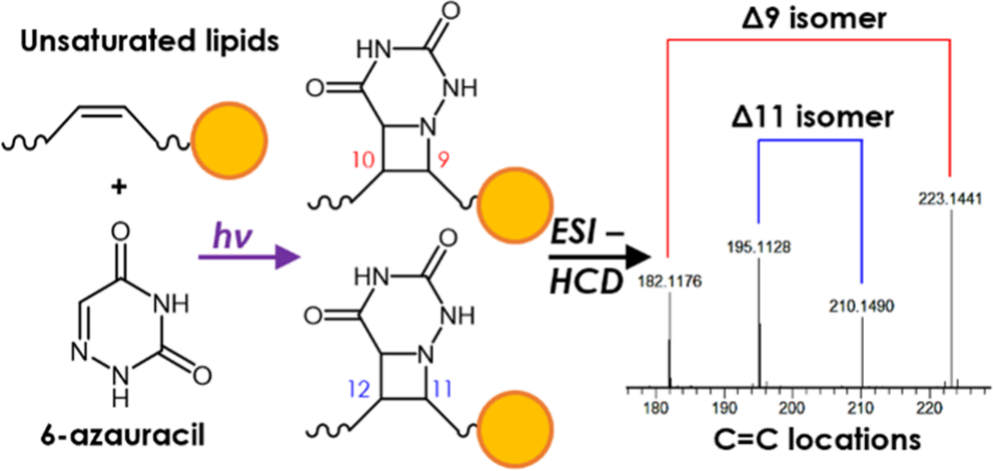

The evaluation of double bond positions in fatty acyl chains has always been of great concern given their significance in the chemical and biochemical role of lipids. Despite being the foremost technique for lipidomics, it is difficult in practice to obtain identification beyond the fatty acyl level by the sole high-resolution mass spectrometry. Paternò–Büchi reactions of fatty acids (FAs) with ketones have been successfully proposed for pinpointing double bonds in FAs in combination with the collision-induced fragmentation technique. In the present paper, an aza-Paternò–Büchi (aPB) reaction of lipids with 6-azauracil (6-AU) was proposed for the first time for the determination of carbon–carbon double bonds in fatty acyl chains using higher collisional dissociation in the negative ion mode. The method was optimized using free FA and phospholipid analytical standards and compared to the standard Paternò–Büchi reaction with acetone. The introduction of the 6-AU moiety allowed enhancing the ionization efficiency of the FA precursor and diagnostic product ions, thanks to the presence of ionizable sites on the derivatizing agent. Moreover, the aPB derivatization allowed the obtention of deprotonated ions of phosphatidylcholines, thanks to an intramolecular methyl transfer from the phosphocholine polar heads during ionization. The workflow was finally applied for pinpointing carbon–carbon double bonds in 77 polar lipids from an yeast (*Saccharomyces cerevisiae*) extract.

Lipidomics is an emerging member of the omics sciences that aims at the qualitative and quantitative determination of the entire set of lipids (the lipidome) in a cell, tissue, or organism.^1^ Lipids are known to play crucial roles in biological systems, as constituents of the cell membranes,^2^ as energy storage compounds,^3^ and for signal transduction,^4^ and an increasing amount of evidence has demonstrated that the dysregulation of lipid metabolism is correlated with the progression of various pathologies.^5−8^

In the latest decades, lipidomics by either shotgun high-resolution mass spectrometry (HRMS) or liquid chromatography coupled to HRMS (LC-HRMS) has been increasingly attracting researchers as a powerful tool for elucidating the composition and alteration of the lipidome.^9,10^ HRMS has emerged as the foremost technique for lipidomics for its high sensitivity and specificity in mixture analysis and for the possibility to perform non-targeted experiments.^11^ A crucial step in any lipidomics analysis is represented by confident lipid identification, which is usually accomplished by the inspection of high-resolution accurate masses and tandem MS (MS/MS or MS^n^) spectra. The degree to which lipid structures can be elucidated is determined by the type and extent of the observed fragment ions.^11^ As such, a hierarchy of lipid identification has been frequently described based on the level of structural detail, from the individuation of the lipid class and sum composition to the identification and localization of the fatty acyl composition and, finally, the evaluation of the stereochemical properties.^12^ In practice, it is difficult to obtain identification beyond the fatty acyl level by the sole HRMS, and even the determination of lipid classes and sum composition is hindered by several phenomena, for example, isomeric mass overlaps.^13,14^ Despite being potentially employed for detailed structural characterization, nuclear Magnetic Resonance (NMR) does not represent a practical alternative to HRMS as it requires relatively large amounts of purified lipid species and time-consuming analysis.

Relevant studies have pointed out the significance of double bond positions on fatty acids (FAs) in the chemical and biochemical roles of lipids.^15,16^ However, the location of C=C bonds cannot be effectively achieved by tandem MS experiments with the common collision-induced dissociation (CID) techniques due to the high bond energy associated with carbon–carbon double bonds.^17^

In the latest years, several innovative approaches for pinpointing carbon–carbon double bonds have been proposed. For example, ozone-induced dissociation (OzID) involves a gas-phase ion/molecule reaction inside the mass spectrometer that results in fragment ions that are diagnostic of the C=C location.^18^ On the other hand, chemical derivatization prior to ionization, for example, epoxidation reactions,^19^ has been proposed as an alternative to OzID with no need for any hardware modification of the mass spectrometer. Paternò–Büchi (PB) reactions with acetone for pinpointing double bonds in FAs have been proposed for the first time by Xia et al.^17,20^ PB reactions are [2 + 2] photocycloadditions between excited carbonyl and alkene groups that generate four-membered oxetanes. PB-derivatized lipids that undergo CID fragmentation could be successfully used for determining the position of C=C bonds. Following the success of PB reactions, several variations have been proposed throughout the years for improving reaction conditions and yields.^21−25^

Despite the large body of literature concerning chemical derivatization by the PB reaction and its variants, most studies were based on CID fragmentation rather than higher-collisional dissociation (HCD).^26−28^ Recently, the group of Heiles has employed MALDI-HCD for carbon–carbon double bond localization.^29^ Moreover, it has been reported that MS/MS spectra of underivatized lipids by HCD are less rich and diagnostic than those obtained by CID. In the case of phospholipids, ions deriving from the polar head by HCD are so stable that often no other information can be obtained.^30^

In the present study, we propose a novel method for pinpointing C=C bonds based on the aza-Paternò–Büchi (aPB) reaction with 6-azauracil (6-AU)^31^ and HCD dissociation in the negative ion mode [ESI(−)], as a complementary alternative to PB reactions that are usually coupled to the positive ion mode [ESI(+)]. Similar to PB reactions, aPB reactions are [2 + 2] photocycloadditions between excited imine and alkene groups that generate four-membered azetidines.^32,33^ 6-AU was chosen for introducing functional groups that could potentially allow enhancing the ionization efficiency of fatty acyl ions both in positive and negative ion modes.^34^ The method was first optimized using free FAs and phospholipid analytical standards and then applied to the characterization of polar lipids in an yeast extract. To the best of our knowledge, the proposed methodology is the first chemical derivatization based on an aPB reaction, which granted feasibility ESI(−) for all analyzed lipid classes.

## Experimental Section

### Lipid Nomenclature

Shorthand notation of the lipid species was based on the guidelines of LIPID MAPS.^35,36^ The location of carbon–carbon double bonds was based on the Δ-nomenclature, in which the carbon atoms are counted from the alpha carbon of the carboxyl/ester group. Underscore (“_”) indicates that the *sn*-position is unspecified.

### Chemicals

Optima mass spectrometry (MS) grade water, acetonitrile (ACN), methanol (MeOH), and isopropanol (i-PrOH) were purchased from Thermo Fisher Scientific (Waltham, MA, United States). Glacial acetic acid, ammonium acetate, 6-AU, oleic acid (18:1 Δ9), vaccenic acid (18:1 Δ11), linoleic acid (18:2 Δ9,12), α-linolenic acid (18:3 Δ9,12,15), γ-linolenic acid (18:3 Δ6,9,12), chloroform, acetone, n-butanol (*n*-BuOH), and phosphoric acid were purchased from Merck (Darmstadt, Germany). 1,2-dioleoyl-*sn*-glycero-3-phospho-(1′-myo-inositol) (PI 18:1/18:1) and 1-palmitoyl-2-oleoyl-*sn*-glycero-3-phosphocholine (PC 16:0/18:1) were purchased from Avanti Polar Lipids (Birmingham, AL, USA). FA stock solutions were prepared in pure MeOH at 100 μmol L^–1^. Phospholipid stock solution were prepared in MeOH/CHCl_3_ 95:5 (*v/v*) at 100 μmol L^–1^. Yeast from *Saccharomyces cerevisiae* was purchased from Merck.

### Yeast Lipid Extraction

The yeast lipidome was extracted, as reported by Khoomrung et al. with minor modifications.^37^ After cell disruption, a Folch lipid extraction was carried out. Briefly, 100 mg of disrupted yeast cells was added to 2.33 mL of MeOH and vortexed for 30 min at room temperature. Later, 4.66 mL of CHCl_3_ was added, and the mixture was kept vortexing for 30 min at room temperature. Finally, 1.7 mL of NaCl (0.73% *w*/*v*) was added into the tube and centrifuged at 2000*g* at 4 °C for 15 min allowing for phase separation. The organic phase was collected and dried up with a Speed-Vac SC 250 Express (Thermo 164 Avant, Holbrook, NY, USA).

### Offline aPB, PB, and Competitive aPB/PB Reaction

Lipid standards and 6-AU were dissolved in 500 μL of MeOH/H_2_O 70:30 (*v/v*) for the aPB reaction with final concentrations of 10 μmol L^–1^ and 1 mmol L^–1^, placed in a quartz cuvette, and purged with nitrogen gas to remove residual oxygen. The cuvette was irradiated at 254 nm using a Spectroline E-series UV lamp with shortwave emission (Thermo Fisher Scientific) for 15 min at room temperature. An offline PB reaction with acetone as a reagent was carried out, as previously reported.^21^ An offline competitive aPB/PB reaction was carried out as follows. Lipid standards (10 μmol L^–1^) and 6-AU (1 mmol L^–1^) were dissolved in 500 μL of acetone/MeOH/water 35:35:30 (*v/v/v*), placed in a quartz cuvette, purged with nitrogen gas to remove residual oxygen, and finally placed in parallel with the UV lamp for 15 min at room temperature. The yeast lipid extract was treated under the same conditions for aPB, PB, and competitive aPB/PB reactions. Reactions mixtures were dried with a Speed-Vac SC 250 Express and finally reconstituted with 100 μL of H_2_O/i-PrOH/n-BuOH (69:23:8, *v/v/v*) with 5 mM H_3_PO_4_ for further HPLC-HRMS analysis. The experiments were repeated in triplicate.

### UHPLC-HRMS Conditions

Lipid separation was carried out by a Vanquish binary pump H (Thermo Fisher Scientific, Bremen, Germany), equipped with a thermostated autosampler and column compartment, on a C8 Hypersyl GOLD (100 × 2.1 mm, 1.9 μm particle size; Thermo Fisher Scientific) at 60 °C with a flow rate of 500 μL min^–1^. The mobile phases consisted of H_2_O/CH_3_COOH (99.85:0.15, *v/v*) with 5 mmol L^–1^ CH_3_COONH_4_ (phase A) and MeOH/*i-*PrOH/CH_3_COOH (79.85:20.00:0.15, *v/v/v*) with 5 mmol L^–1^ CH_3_COONH_4_ (phase B). The chromatographic gradient was as follows: 5 min at 30% phase B, from 30 to 99% phase B in 15 min, 99% phase B for 10 min (washing step), 99 to 30% phase B in 1 min, and 30% phase B for 5 min (equilibration step). The injection volume was 10 μL.

The HPLC system was coupled to the Q Exactive hybrid quadrupole-Orbitrap mass spectrometer (Thermo Fisher Scientific) with the following source settings: spray voltage 3.5 kV [ESI(+)) and 2.5 kV (ESI(−)]; capillary temperature 275 °C [ESI(+)] and 320 °C [ESI(−)]; sheath gas flow rate 55 [ESI(+)] and 35 [ESI(−)] arbitrary units (a.u.); auxiliary gas flow rate 15 a.u.; auxiliary gas heater temperature 450 °C [ESI(+)], and 400 °C [ESI(−)]. An exclusion list containing the most intense ions detected in a blank sample consisting of H_2_O/*i*-PrOH/*n*-BuOH (69:23:8, *v/v/v*) with 5 mM H_3_PO_4_ was added to the mass-spectrometric method. Full-scan MS data were acquired in the range of 200–1200 *m/z* with a resolution (full width at half-maximum, fwhm) of 70,000. The isolation window width was 2 *m*/*z*. TOP 5 data-dependent acquisition (DDA) MS/MS fragmentation was performed with a resolution (fwhm) of 35,000. AGC target value was set at 100,000, and dynamic exclusion was set to 3 s. Collision energy fragmentation was achieved in the HCD cell at 40 NCE for FA standards and 30 NCE for polar lipids. Raw data files were acquired by Xcalibur software (version 3.1, Thermo Fisher Scientific). All samples were run in triplicate.

### Lipid Identification

Yeast lipidome was identified using LipidSearch software (Thermo Fisher Scientific) with the following parameters: HCD fragmentation was selected; exp type LC–MS; precursor and parent tolerance 5.0 and 8.0 ppm; and target class FAs, phospholipids, and sphingolipids. Details are reported in Table S1. aPB and PB reaction products were manually searched in the MS data considering the relative mass shifts (+113.0225 for aPB and +58.0419 for PB).

## Results and Discussion

### aPB Reaction with 6-AU

In the field of organic synthesis, the aPB reaction is far less developed compared to the PB reaction to generate oxetanes, despite the great interest in the synthesis of azetidines.^32^ Unlike alkenes and ketones, in fact, the excited state of imines is susceptible to radiationless decay back to the ground state that makes them traditionally unreactive in [2 + 2] photocycloadditions.^38^ As a result, few imine scaffolds have been reported to successfully participate in aPB reactions.^32^ Among these, 6-AU has been successfully used for [2 + 2] photocycloadditions with alkenes and alkynes.^31,32^ 6-AU had several characteristics that made it a promising candidate for the aim of obtaining fatty acyl derivatives with a high ionization efficiency and stability. First, it reacted under mild conditions in combination with ethylene, which is a non-activated alkene resembling the non-activated C=C bonds of FAs. Moreover, 6-AU presented a relatively high molecular weight (113.0225), an imine group that would have turned into a strongly alkaline tertiary amine following the aPB reaction and an imide group with an acidity comparable to phenol.^39^

The aPB reaction of 6-AU was first tested on the fatty acyl standards mentioned in the Chemicals section for the evaluation of the reaction and instrumental conditions as well as the HCD fragmentation pathways of the reaction products. The work of Aitken was used as a trace,^31^ and MeOH and ACN were compared, with the latter providing a negligible amount of reaction products despite having been used for other aPB reactions.^33^

For the evaluation of the reaction time, FA 18:1 Δ9 and FA 18:1 Δ11 were dissolved in MeOH/H2O 70:30 (v/v) with 6-AU and kept for 1, 2, 5, 10, 15, 30, and 60 min under UV radiation. The reaction mixtures were analyzed by LC–MS, and the total peak areas of *m/z* 394.2696 (6-AU derivative) and 297.2429 (oxidized FA 18:1) were evaluated (Figure S1). For both FAs, a rapid increase of *m/z* 394.2629 was observed in the first 15 min, whereas a much less rapid growth was observed at 30 and 60 min. The oxidized FA 18:1, on the other hand, increased slower in the first 10 min and then started increasing at a steady pace. For these reasons, 15 min was chosen as the optimum reaction time. In our peculiar application of the aPB reaction, yields were not the prime aim, and the reaction time was evaluated as a compromise between the need for relatively fast reactions and a good instrumental response. However, the decrease in abundance of the precursor was calculated by comparing the areas of the underivatized precursor of the reactions and those of the control reaction (analogue reaction mixtures that were not subject to UV emission). The underivatized precursor was found to be about 59–69% without any particular trend observed among the several analyzed FAs. Based on the 3:1 ratio between the aPB derivatives and the oxidized FAs at 15 min(Figure S1), the reaction yield can be hypothesized at 23–30%, keeping in mind that this calculation is a possible overestimation due to other possible minor reaction byproducts^21^ and because the aPB or collateral reactions could have a significant repercussion on the ionization efficiency.

**Figure 1:**
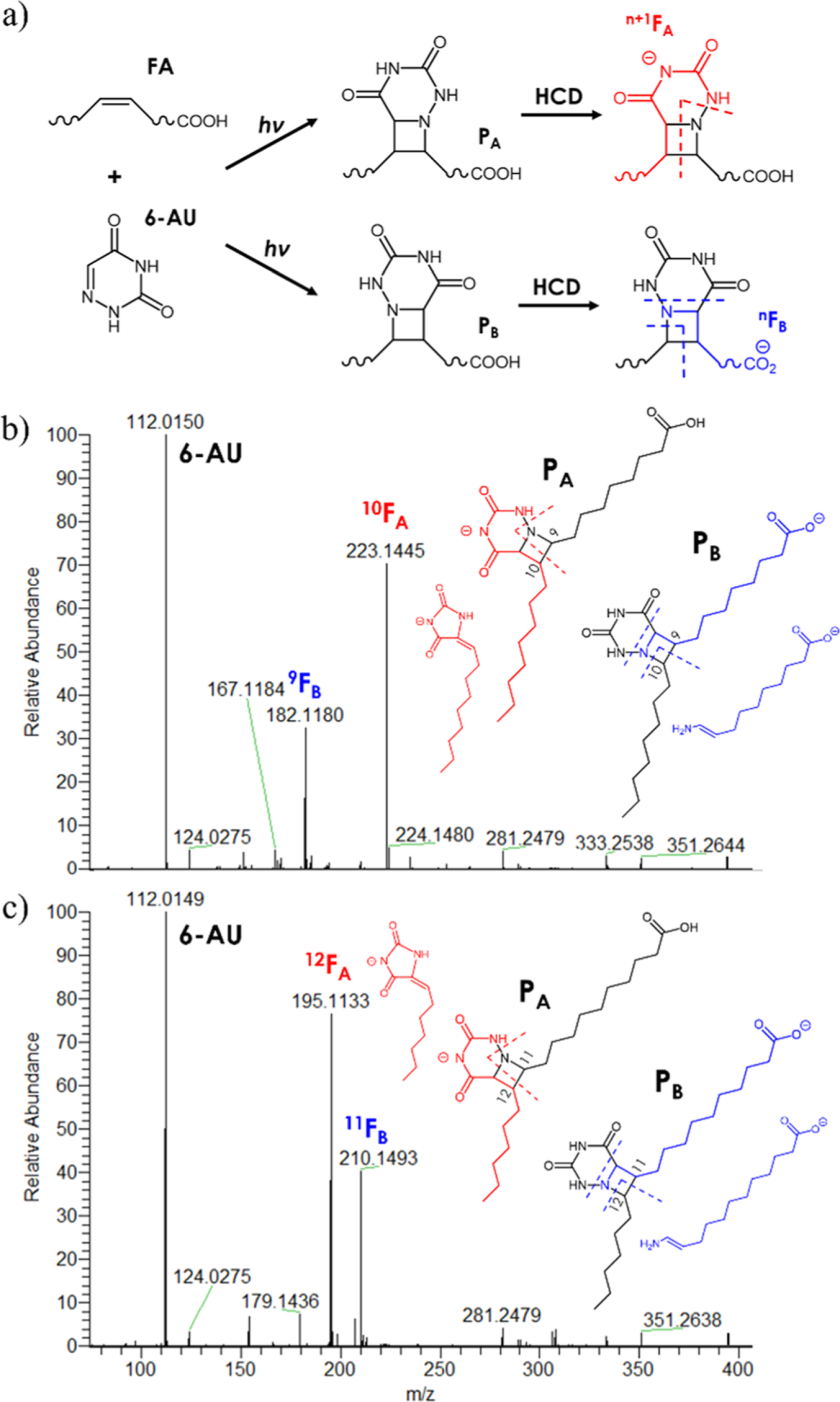
a) Schematic representation of the aPB reaction and obtention of the diagnostic ions following HCD fragmentation; (b) HCD MS/MS spectrum of FA 18:1 Δ9 following the aPB reaction; and (c) HCD MS/MS spectrum of FA 18:1 Δ11 following the aPB reaction.

For the prochirality of fatty acyl double bonds, several stereoisomeric products and, subsequently, broad chromatographic peaks were obtained after LC–MS analysis, in analogy with the previous results by Zhao for the PB reaction.^21^ Regardless of the stereochemical properties, two main reaction products were expected (P_A_ and P_B_, Figure 1a).

The complete MS/MS spectrum of the 6-AU derivative of FA 18:1 comprised, beyond the ion of 6-AU (112.0147), two main ions in the negative ion mode [ESI(−)]; at this stage, ESI(−) was preferred to ESI(+) for the clearer obtained spectra. The two main ions of the MS/MS spectrum (labeled F_A_ and F_B_, Figure 1a) were rationalized as deriving from either P_A_ or P_B_ with the hypothesized structures shown in Figure 1b,c and furnished information on the two positions of the original C=C bond; therefore, the two product ions were termed ^n+1^F_A_ and ^n^F_B_, with n being the position of the double bond based on the Δ-nomenclature (Figure 1a). In Figure 1b,c, the HCD MS/MS spectra of FA 18:1 Δ9 (oleic acid) and FA 18:1 Δ11 (vaccenic acid) are shown as representative examples. The broad peaks obtained by LC separation of the aPB derivatives helped evaluate the fragmentation patterns;^21^ in fact, the inspection of single MS/MS scans allowed observing the MS/MS fragmentation of either P_A_ or P_B_ individually (in any other case, the average MS/MS spectra were inspected). In Figure S2, MS/MS single scans that were putatively associated with the *P*_A_ of FA 18:1 Δ9 (a) and 18:1 Δ11 (b) are shown. Other than the base peak corresponding to the F_A_ ion, the complementary ion is visible in the spectrum, thus confirming the fragmentation pathway shown in Figure 1a. In comparison to the fragmentation pathway of the PB reaction, in which all product ions had a negative charge on the carboxyl group,^20^ the acidity of the imide group allowed obtaining fragments from the other side of the double bond (F_A_) (Figure S3). Figures S4–S6 show the HCD MS/MS spectra of the aPB derivatives of FA 18:2 Δ9,12 (linoleic acid), FA 18:3 Δ9,12,15 (α-linolenic acid), and FA 18:3 Δ6,9,12 (γ-linolenic acid). Not unexpectedly, for each double bond two main reaction products and, subsequently, two main product ions were observed (a total of four diagnostic ions in the case of FA 18:2 and six in the case of FA 18:3).

Relative quantitation based on diagnostic ion intensities was tested with a series of mixtures of FAs 18:1 Δ9 and Δ11, with the total concentration kept constant (20 μmol L^–1^) while the molar ratios (Δ9/Δ11) varied from 50 to 0.5, in agreement with Δ9 being the major isomer. The diagnostic ions were employed for relative quantitation since the precursor ions coeluted and had the exact same *m/z*. The ion intensities I_AB_ calculated as a sum of F_A_ and F_B_ of each isomer (I_9AB_/I_11AB_) were plotted against the concentration ratios (Figures S7a and S8). Good linearity (*R*^2^ = 0.9991) and a wide dynamic range (from 49:1 to 1:2) were obtained. However, given the major abundance of F_A_-type ions (ca. 2:1 compared to F_B_-type ions), a second plot was obtained considering the sole contribution of F_A_ ions (I_9A_/I_11A_) (Figure S7b). Considering that the second plot furnished even better results (*R*^2^ = 0.9995), for polyunsaturated α- and γ-linolenic FAs, the experiment was repeated by considering the sole contribution of F_A_-type ions in the molar ratio range from 10:1 to 1:10 (Figure S9). The presence of three pairs of reaction products, in fact, lowers the ion intensities of the diagnostic peaks that made F_B_-type ions occasionally not detectable at the extremes of the considered molar ratio range.

### Comparison of PB and aPB Reaction Products by ESI(−) HCD

To assess the advantages and disadvantages of the aPB reaction in an HCD-based lipidomics experiment, a comparison with a classical PB reaction with acetone as well as a competitive aPB/PB reaction were carried out on FA and phospholipid standards. Acetone was chosen since it represents the simplest and more widespread PB reagent^40^ and since it does not carry out functional groups that could affect the ionization efficiency in ESI(−). Acetylpyridine is nowadays considered the most efficient PB reagent, but its use has been limited to ESI(+),^26,27,41^ possibly due to the pyridyl functional group that enhances the ionization efficiency in ESI(+). It is important to note from the outset that, unlike acetone, 6-AU carries out functional groups that are supposed to enhance the ionization efficiency of the diagnostic production ions in ESI(−). At present, several PB reagents with functional groups that could enhance the ionization in ESI(+) have been proposed.^26^ On the other hand, PB reagents that are specifically selected to boost ionization in ESI(−) are currently missing, despite possibly furnishing similar advantages to aPB derivatization with 6-AU.

It is well established that CID is a more gentle fragmentation technique than HCD. In the field of proteomics, HCD has been demonstrated to furnish richer spectra that provide higher search engine scores than CID.^42^ Conversely, less rich MS/MS spectra are obtained for polar lipids, for example, in the case of phosphatidylcholines (PCs) and sphingomyelins, MS/MS spectra in ESI(+) show generally the sole peak of the headgroup (*m*/*z* 184.0731). Few papers have dealt with the comparison of CID and HCD in lipidomics;^43^ however, on the HRMS spectral database mzCloud (https://www.mzcloud.org/), CID and HCD spectra of PI (16:0/16:0) in ESI(−) and PC (16:0/16:0) in ESI(+) are available. In both cases, HCD spectra are less diagnostic and comprise mostly (if not exclusively) product ions deriving from the polar headgroups regardless of the value of NCE.

**Figure 2:**
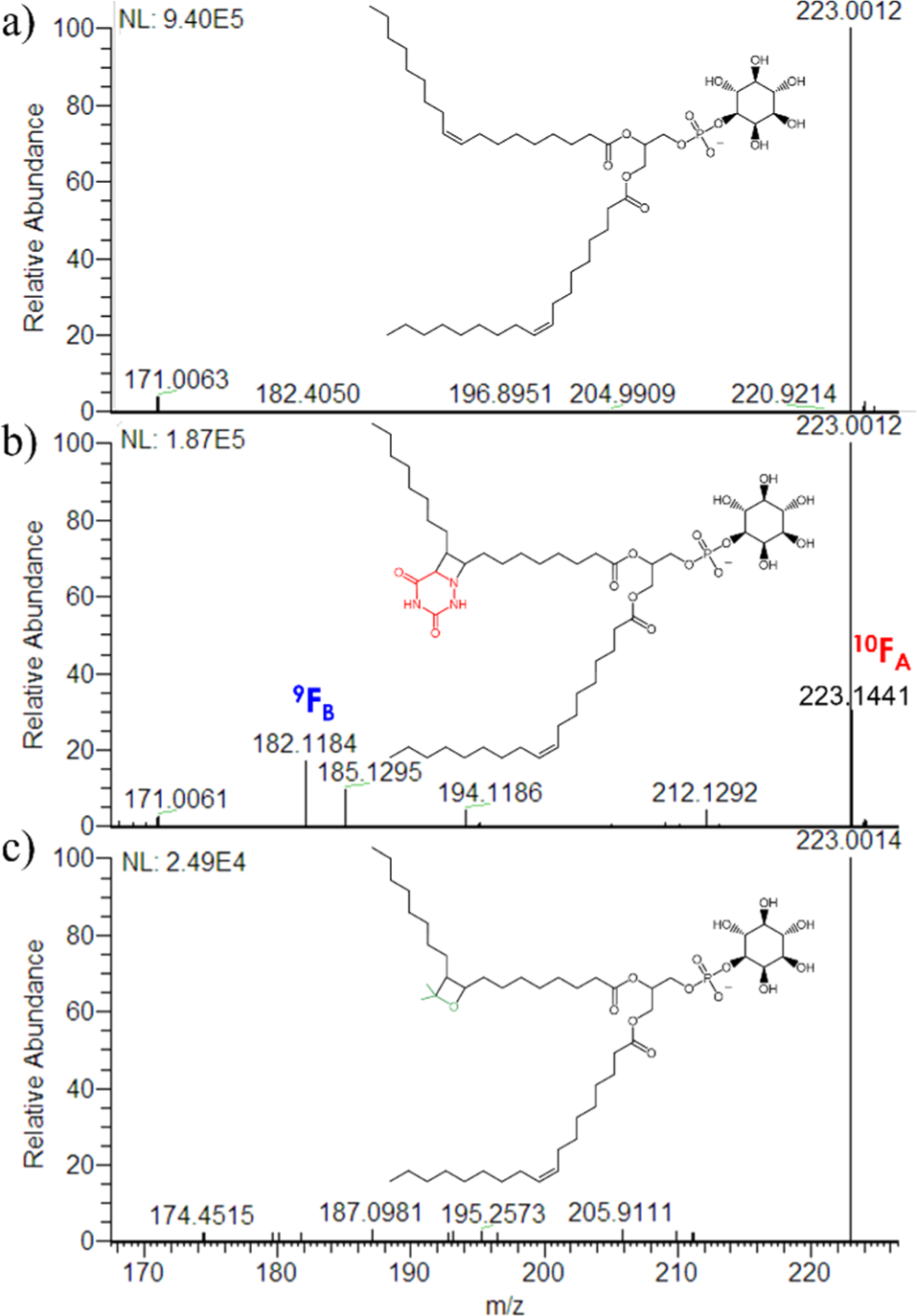
HCD MS/MS spectrum (30 NCE) in the negative ion mode and the range 170–230 *m*/*z* of (a) underivatized PI (18:1 Δ9/18:1 Δ9); (b) aPB derivative of PI (18:1 Δ9/18:1 Δ9) with 6-AU; and (c) PB derivative of PI (18:1 Δ9/18:1 Δ9) with acetone.

At present, few studies have coupled PB reactions and HCD,^26−29,44,45^ and CID was preferred even when HCD-based facilities were available.^46^ Moreover, in all but one case in which rhamnolipid precursors were analyzed,^45^ ESI(+) was preferred over ESI(−). PB reactions carried out on FAs were analyzed by LC-HRMS, and HCD fragmentation at 40 NCE in ESI(−) furnished the analogue cleavage pathways that were described by Ma,^20^ with an intense base peak of the deprotonated PB derivative, the ion of the underivatized FA, and the two diagnostic fragments (Figure S10). On the other hand, PB derivatization coupled with HCD was found unsuitable for the evaluation of the double bond position on the PI analytical standard in ESI(−). The derivatives of PI (18:1 Δ9/18:1 Δ9) obtained by aPB and PB reactions were analyzed by LC-HRMS in ESI(−) and subject to HCD fragmentation. Collision energy of 30 NCE was preferred to 40 NCE because it allowed obtaining product ions deriving from the cleavage of the glycerol ester bond. In Figure 2, the HCD MS/MS spectra are reported for the underivatized PI (a), its aPB derivative (b), and the PB derivative (c) in the range 170–230 *m*/*z* (spectra in the whole range are reported in Figure S11). The PB derivative was almost identical to the underivatized PI, meaning that the pair of diagnostic ions (*m/z* 171.1025 and 197.1545) had a significantly lower ionization efficiency than the ions deriving from the polar headgroup (e.g., *m/z* 223.0012). The aPB derivative, on the other hand, allowed visualizing the two diagnostic ions, ^10^F_A_ at *m/z* 223.1441 and ^9^F_B_ at *m/z* 182.1184, that were also generated by the cleavage of FA 18:1 Δ9, as shown in Figure 1a. As shown in Figure S11b,c, no diagnostic ions were present in these experimental conditions at higher *m*/*z* deriving from to the cleavage of the four-membered ring before the cleavage of the glycerol-FA bond. In Figures 2 and S11, the ion intensities (expressed in NL by Xcalibur software) of the MS/MS spectra are shown.

The intensity of the aPB reaction product was about 7.5 times higher than the PB product for PI (18:1 Δ9/18:1 Δ9) in ESI(−), a result that was consistent with the total ion current (TIC) of the MS spectrum and confirmed by the FA standard (3–15 times higher). A competitive aPB/PB reaction of PI (18:1 Δ9/18:1 Δ9) was carried out in 500 μL of acetone/MeOH/H_2_O (35:35:30, *v/v/v*) and 6-AU 1 mmol L^–1^. The solvent mixture was a compromise for allowing the solubility of 6-AU and the standard PI and at the same time avoiding ACN that was found unsuitable for the aPB reaction. Despite the extremely higher concentration of acetone (about 5 mol L^–1^) compared to 6-AU, the preference for the aPB reaction was even more pronounced. In Figure S12, the TIC of the *m/z* of the underivatized PI, as well as the aPB and PB derivatives are shown after the integration of the peak areas. In these experimental conditions, the aPB derivative had a total peak area 45 times higher than the corresponding PB derivative. There are several explanations for these results. Acetone is itself a triplet sensitizer and has been even employed in some aPB reaction procedures for acetone-sensitized excitation.^32^ Moreover, the lower yield of the PB reaction products can be attributed to the quenching of the triplet state of acetone. Previous studies have demonstrated that protic solvents and amines are responsible for quenching the triplet state of acetone. In particular, uracil (which is very similar to 6-AU) can quench the triplet state of acetone with a constant rate that is more than 3 orders of magnitude higher than methanol.^47^ As such, a proper competitive reaction cannot be achieved. However, the triplet sensitizer behavior of acetone and the scarce contribution of PB byproducts could potentially be of great interest for future application of the aPB reaction in lipidomics.

### Characterization of Unsaturated Lipids in an Yeast Extract

For evaluating the efficacy of the proposed methodology on a real–world complex matrix, a lipid extract was obtained from yeast (*S. cerevisiae*) and subject to aPB derivatization. Yeast was chosen since it represents a model system for the eukaryotic cells, in which most lipid classes are present.^48,49^ Before derivatization, the yeast lipid extract was first analyzed by LC-HRMS and data analysis by LipidSearch software. After manual annotation of the annotated lipids, their corresponding aPB derivatives were manually searched in the raw data files. Table S2 summarizes the results: for each lipid sum composition, the underivatized and the aPB derivatives are shown, alongside the retention times, proposed formulas, adducts, experimental and calculated *m/z*, Δmass, and the diagnostic product ions of both species. A total of 47 sum compositions and 77 unsaturated lipid species were annotated from the yeast extract with the information of the double bond location, a significantly improved result compared to that of Ma^17^ using the PB reaction with acetone on the same matrix (19 unsaturated lipids). In agreement with the previous findings on yeast,^48,49^ PCs (23 unsaturated lipids), PIs (22), and PEs (17) were the most abundant lipid classes, followed by FAs (7), PSs (6), and PGs (2), and the fatty acyl chains comprised mostly saturated and monounsaturated species, with minor abundances of polyunsaturated linoleic acid. FA 17:1, 18:2, and 20:1 presented a single double bond location; on the other hand, both FA 16:1 and 18:1 were mixtures of the Δ9 and Δ11 isomers. Based on the previous results on relative quantitation of the double bond isomers, an amount of 2.4 and 7.1% of Δ11 isomer was calculated for FA 16:1 and 18:1, respectively. Among the identified phospholipids, several sum compositions presented FA 18:1 Δ11 isomers in the range of 1.5–5.5% (Table S2). Interestingly, FA 16:1 Δ11 was never found on a phospholipid backbone, despite the high abundance of palmitoleyl chains.

**Figure 3:**
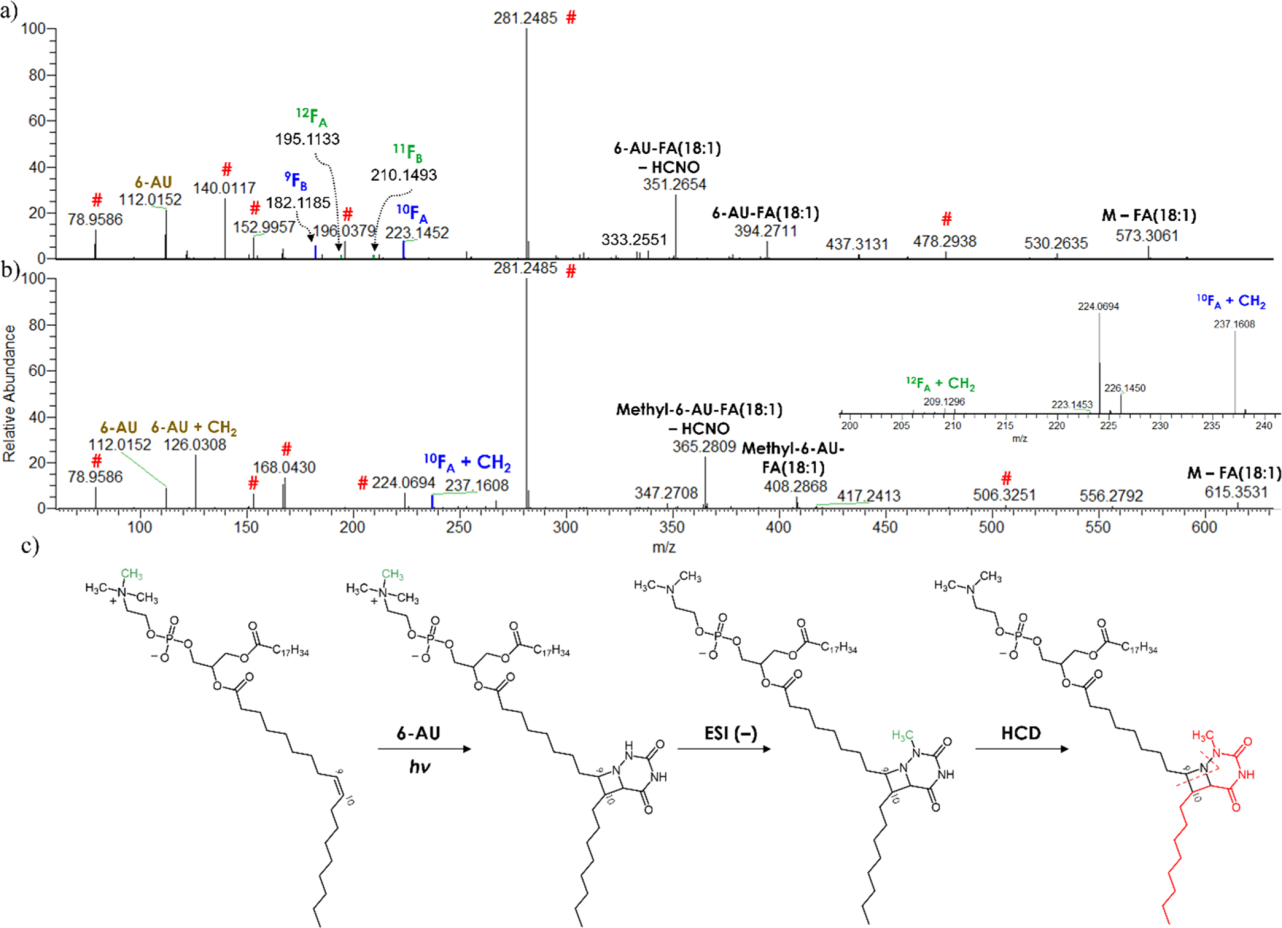
HCD MS/MS spectrum (30 NCE) in the negative ion mode of (a) deprotonated aPB derivative of sum composition PE (36:2) and (b) deprotonated aPB derivative of sum composition PC (36:2). (c) Hypothesized reaction, ionization, and fragmentation mechanisms of PCs involving a methyl rearrangement on the 6-AU moiety. The product ions marked with # also appear in the MS/MS spectrum of the underivatized species (Figure S13b).

In Figure 3a, the MS/MS HCD spectrum of the aPB-derivatized sum composition PE (36:2) is shown, while Figure S13a shows the MS/MS HCD spectrum of the underivatized lipid species. In the spectrum of the derivatized species, the product ions marked with a hash (#) are in common with the underivatized species and allowed confirming the origin of the aPB derivative. The other product ions derived mostly from the derivatized fatty acyl chain: *m/z* 573.3061 indicates a loss of underivatized FA 18:1; *m/z* 394.2711 and 351.2654 are the ions of the 6-AU derivative of FA 18:1; *m/z* 223.1452 and 182.1185 are the diagnostic ions of FA 18:1 Δ9 (Figure 1a); *m/z* 210.1493 and 195.1133 are the diagnostic ions of FA 18:1 Δ11 (Figure 1b); and *m/z* 112.0152 is the deprotonated 6-AU. The evaluation of the relative intensities of the diagnostic ions allowed estimating about 4% of Δ11 isomer (Table S2). All other derivatized phospholipids were annotated with the same rationale.

### Case of Derivatized Phosphatidylcholines

PCs present a zwitterionic structure with a negatively charged phosphate group and a positively charged quaternary ammonium. The peculiar structure of PCs hinders their deprotonation when subject to ESI ionization, and in-source fragmentation of the ammonium group or its interaction with negatively charged ions represents the most abundant ionization pathways.^14^Figure S14a–c shows the total ion currents of the *m/z* corresponding to the adducts [M—CH_3_]^−^, [M—H]^−^, and [M + CH_3_COO]^−^ of PC (16:0/18:1) after PB reaction with acetone. None of the three *m/z* showed a significant peak, whereas the corresponding protonated adduct (Figure S14d) had a much higher ionization efficiency than the negative counterparts. However, the MS/MS spectrum of the PB derivative of PC (16:0/18:1 Δ9) was non-diagnostic for pinpointing carbon–carbon double bonds (Figure S15b), with apparently no differences compared to the spectrum of the underivatized PC (Figure S15a). This result marked a great inconsistency with the spectrum of a similar compound that was reported by Ren,^27^ in which two diagnostic ions were present at *m*/*z* 650 and 676. This inconsistency was attributed to the low abundance of the diagnostic ions (lower than 2:1000 compared to the base peak) that require higher concentrations, as demonstrated by the MS/MS spectrum of the PB derivative of high-abundance PC (16:1/16:1) from the yeast extract (Figure S16), in which the diagnostic ions are effectively visible. This result is in agreement with HCD furnishing less diagnostic spectra than CID at the high *m*/*z* range.^30^

Interestingly, the aPB reaction product of PC (16:0/18:1) showed an intense ion corresponding to the deprotonated adduct (Figure S14e), whereas the two typical adducts of underivatized PCs were negligible. As such, the presence of the acid imide group on the 6-AU moiety was thought to allow bypassing the structural limitations of PCs in ESI(−) that result in the generation of more than one adduct, a subsequent loss of sensitivity, and several isomeric mass overlaps that hinder the determination of molecular formulas and mass compositions.^10^

Nevertheless, a careful inspection of the MS/MS spectra of aPB derivatives of PCs supported a different possible mechanism of ionization. In Figure 3b, the HCD MS/MS spectrum of the sum composition PC (18:1/18:1) from the yeast extract is shown and compared to that of PE (18:1/18:1). PC (18:1/18:1) was selected for easier comparison with the PE analogue, but the same rationale applies to standard PC (16:0/18:1) (Figure S17). Similar to PE (18:1/18:1) that was described in the previous paragraph, the ions marked with the hash were common to the corresponding underivatized PC (Figure S13b) and allowed confirming the lipid precursor. However, all ions that could have possibly derived from the derivatized FA of the PC showed an increase of about 14.0156 (a methylene unit) compared to the corresponding PE. As no ions indicating longer fatty acyl chains were present, the mass shift was attributed to the derivatized part of the molecule; moreover, *m/z* 615.3531 corresponds to the loss of a FA 18:1 from the aPB-derivatized precursor ion, in analogy with *m/z* 573.3061 in the case of the PE. Thus, the mass shift was rationalized as a methyl rearrangement from the quaternary ammonium to the 6-AU moiety (Figure 3c). The methyl rearrangement is in agreement with the absence of in-source fragmentation during the ionization of the aPB derivative and with the product ions shown in Figure 3b. In particular, according to the hypothesized fragmentation pathways (Figure 1a), the presence of a diagnostic F_A_-type ion with a 14.0156 mass shift is in agreement with the described rearrangement. The final proof for this mechanism is represented by the presence of *m*/*z* 126.0308, which corresponds to the sum composition of a methylated 6-AU. Intermolecular methyl transfers from the quaternary ammonium have been previously reported by Zhang^50^ for anionic adducts of PCs with negative ions and recently confirmed by Deng,^46^ which inspected the MS behavior of bicarbonate adducts of PCs. These rearrangements were rationalized based on a nucleophile substitution by the negative ion on one of the methyl groups, a mechanism that could explain the intramolecular methyl rearrangements observed in the aPB derivatives of PCs.

Once the methyl rearrangement was kept in mind, the inspection of the MS/MS spectra of aPB derivatives of PCs was straightforward. Given the linear regression shown in Figure S6b, the sole F_A_-type ions were considered for the relative quantitation of fatty acyl isomers in PCs reported in Table S2.

### aPB Derivatives in the Positive-Ion Mode

So far, the HCD MS/MS spectra of aPB derivatives of FAs and polar lipids have been only described in ESI(−), which was proven able to pinpoint carbon–carbon double bonds in HCD-based experiments. Nevertheless, 6-AU allowed also ionizing lipid derivatives in ESI(+). In general, FAs and several classes of polar lipids, for example, PIs, are solely analyzed in ESI(−) due to the complete absence of protonable sites.^11^ The aPB derivatives of FAs were as effectively ionized in ESI(+) (as in-source fragments after the loss of H_2_O from the carboxyl group) as in ESI(−) (Figure S18), a remarkable result considering that ESI(+) is usually blind to underivatized FAs. In contrast with the clear MS/MS spectra in ESI(−), the ones in the positive mode were much more complex, with a series of neutral losses (H_2_O, CO, HCNO, and NH_3_) and short-chain carbocationic ions. Despite the complexity of these spectra, diagnostic ions could be distinguished for FA 18:1 Δ9 (*m/z* 192.1756) and FA 18:1 Δ11 (*m/z* 164.1442) (Figure S19). Unsurprisingly, the ions derived from the non-carboxyl side of the double bond of each FA.

MS/MS spectra of aPB-derivatized standard phospholipids and yeast lipids were also evaluated. PI (18:1/18:1) confirmed the good enhancement of the ionization efficiency that was observed for the FA standards. In the HCD MS/MS spectrum of the PI, sequential losses of inositol phosphate, 6-AU, and FA 18:1 were easily distinguished. Moreover, ions of the aPB-derivatized FA 18:1 were present, solving the issue of ESI(+) not furnishing information on the fatty acyl chains when coupled to HCD. Finally, in the lower *m/z* range, a minor abundance of the diagnostic ion at *m/z* 192.1756 was found, proving the ESI(+) is also feasible for pinpointing carbon–carbon double bonds in phospholipids after aPB derivatization with 6-AU (Figures 20a). The inspection of the HCD MS/MS spectrum of the PE analogue from yeast furnished the same fragmentation pathways (Figure S20b). However, in the case of PCs, the extremely high ionization efficiency of the phosphocholine headgroup (*m/z* 184.0731) minimized the abundance of the other ions (Figure S20c). It is worth pointing out that the fragmentation pathways of aPB-derivatized lipids with HCD were completely different from those of PB derivatized lipids.^20^ In general, despite effectively being able to enhance the ionization efficiency and, in most cases, to furnish information on the double bond location of FAs and polar lipids, aPB derivatization with 6-AU was way more suitable in ESI(−), which provided much clearer spectra and higher ionization efficiency of the diagnostic ions.

## Conclusions

The determination of the double bond position in fatty acyl chains is one of the most interesting current research areas in MS-based lipidomics. The aPB reaction of lipids with 6-AU was proven to be effective with HCD-based instrumentation in ESI(−). Thanks to the peculiar physicochemical properties of 6-AU, the ionization efficiency of diagnostic fragment ions was boosted; moreover, it allowed the obtention of deprotonated adducts of PCs, overcoming the limitations caused by isomeric mass overlaps in the determination of this class of compounds, thanks to an intramolecular methyl transfer from the phosphocholine head to the 6-AU moiety. In general, the preference of aPB-derivatization with 6-AU for ESI(−) can be considered complementary to the PB-derivatization, which in turn has been proven to perform better in ESI(+).

However, there are still several limitations to overcome. First, the duration of the aPB reaction (15 min) makes it incompatible with the setup of online derivatization workflows. In addition, the labile nature of 6-AU under ESI(+) conditions made it scarcely applicable compared to ESI(−). Under all circumstances, the inherent asymmetry of imines (the same applies to PB and ketones) would always generate multiple reaction products that diminish the abundance of the diagnostic ions.

The use of MS-compatible photosensitizers could help improve the reaction rates, and larger and more stable derivatives of 6-AU could effectively enhance the abundance of the diagnostic ions in ESI(+). Reactions of unsaturated lipids with other imine structures might also be explored. The present study opens up unraveled paths in the determination of the double bond positions in lipidomics in Orbitrap HRMS without the need for extensive sample preparation or instrument modifications.

## Supporting Information Available

Detailed LipidSearch parameters, dispersion graphs of the aPB reaction over time, PB and aPB reaction schemes, TIC and HCD MS/MS spectra, and linear regressions (XLSX)
Polar lipid identification details from the yeast extract following aPB reactions, comprising lipid identity and double bond location, RT, proposed molecular formula, molecular weight, adduct, mass error (ppm), experimental *m*/*z*, calculated *m*/*z*, mass error (ppm), and major product ions (PDF)
