# Supplementary material for: Novel Aza-Paternò-Büchi Reaction Allows Pinpointing Carbon–Carbon Double Bonds in Unsaturated Lipids by Higher Collisional Dissociation
Source: Anal Chem. 2022 Sep 19;94(38):13117–25. doi: 10.1021/acs.analchem.2c02549 (PMC9523615; doi:10.1021/acs.analchem.2c02549)
Supplement: Supplementary file 2 — ac2c02549_si_002.pdf [file ac2c02549_si_002.pdf]

Supporting Information – Word File

A novel aza-Paternò-Büchi reaction allows pinpointing carbon-carbon double bonds in unsaturated lipids by higher collisional dissociation

Andrea Cerrato, Anna Laura Capriotti*, Chiara Cavaliere, Carmela Maria Montone, Susy Piovesana, Aldo Laganà

Department of Chemistry, Sapienza University of Rome, Piazzale Aldo Moro 5, 00185 Rome, Italy

Table of Contents

Table S1. Detailed LipidSearch parameters for polar lipids data processing.

Figure S1. Dispersion graphs for FA 18:1 Δ9 (a) and FA 18:1 Δ11 (b) showing the total peak areas of *m/z* 394.2696 (6-AU derivative) and *m/z* 297.2429 (oxidized FA 18:1).

Figure S2. MS/MS scan associated to the aPB derivative P_A of FA 18:1 Δ9 (a) and FA 18:1 Δ11 (b). Other than the main F_A ion (base peak), the complementary ion is visible in the spectrum (m/z 170.1181 for 18:1 Δ9 and m/z 198.1489 for 18:1 Δ11)

Figure S3. Comparison of the fragmentation pathways that lead to the diagnostic product ions between aPB reaction with 6-AU (a) and PB reaction with acetone (b).

Figure S4. ESI(−)-HCD MS/MS spectrum (40 NCE) of deprotonated FA 18:2 Δ9,12 after photochemical reaction with 6-AU.

Figure S5. ESI(−)-HCD MS/MS spectrum (40 NCE) of deprotonated FA 18:3 Δ9,12,15 after photochemical reaction with 6-AU.

Figure S6. ESI(−)-HCD MS/MS spectrum (40 NCE) of deprotonated FA 18:3 Δ6,9,12 after photochemical reaction with 6-AU.

Figure S7. Linear relationship between the molar ratios of FA 18:1 Δ9 and Δ11 and: (a) the intensities of the diagnostic ion pairs (I_{9AB}/I_{11AB}), (b) the intensities of the diagnostic F_A ions (I_{9A}/I_{11A}).

Figure S8. ESI(−)-HCD MS/MS spectrum (40 NCE) of FA 18:1 Δ9 and Δ11 at equal molar ratio after aPB derivatization.

Figure S9. Linear relationship between the molar ratios of FA 18:3 Δ9,12,15 and Δ6,9,12 and the intensities of the diagnostic F_A ions ($I_{9,12,15A}/I_{6,9,12A}$).

Figure S10. ESI(−)-HCD MS/MS spectrum (40 NCE) of (a) deprotonated FA 18:1 Δ9 and (b) deprotonated FA 18:1 Δ11 after PB derivatization with acetone. The reported nomenclature of the product ions is that of Ma (https://doi.org/10.1073/pnas.1523356113), with the two ions deriving from each of the two main PB products.

Figure S11. ESI(–)-HCD MS/MS spectrum (30 NCE) of a) underivatized PI (18:1/18:1); b) aPB derivative of PI (18:1/18:1); c) PB derivative of PI (18:1/18:1).

Figure S12. Total ion current of a) deprotonated underivatized PI (18:1/18:1); b) deprotonated aPB derivative of PI (18:1/18:1); c) deprotonated PB derivative of PI (18:1/18:1) obtained by the competitive aPB/PB reaction.

Figure S13. ESI(–)-MS/MS spectrum (30 NCE) in negative ion mode of a) underivatized deprotonated PE (18:1/18:1); b) underivatized acetate adduct of PC (18:1/18:1).

Figure S14. Total ion current of the *m/z* corresponding to a) the deprotonated PB derivative of PC (16:0/18:1); b) the demethylated PB derivative of PC (16:0/18:1); c) acetate adduct of the PB derivative of PC (16:0/18:1); d) the protonated PB derivative of PC (16:0/18:1); e) the deprotonated aPB derivative of PC (16:0/18:1).

Figure S15. ESI(+)-HCD MS/MS spectrum (30 NCE) of (a) protonated underivatized PC (16:0/18:1) and (b) protonated PB-derivatized PC (16:0/18:1).

Figure S16. ESI(+)-HCD MS/MS spectrum (30 NCE) of protonated PB-derivatized PC (16:1/16:1) from the yeast extract. A zoom on the high m/z range shows a low abundance of the typical diagnostic peaks for pinpointing carbon-carbon double bonds.

Figure S17. ESI(–)-HCD MS/MS spectrum (30 NCE) in negative ion mode of a) underivatized acetate adduct of standard PC (16:0/18:1); b) deprotonated aPB derivative of standard PC (16:0/18:1).

Figure S18. Total ion current of the *m/z* corresponding to a) the protonated in-source fragment of the aPB derivative of FA 18:1 Δ9; b) the protonated in-source fragment of the aPB derivative of FA 18:1 Δ11; c) the deprotonated aPB derivative of FA 18:1 Δ9; d) the deprotonated aPB derivative of FA 18:1 Δ11.

Figure S19. ESI(+)-HCD MS/MS spectrum (30 NCE) of a) the protonated aPB derivative of FA 18:1 Δ9; b) the protonated aPB derivative of FA 18:1 Δ11. The diagnostic ions are marked with a red circle.

Figure S20. ESI(+)-HCD MS/MS spectrum (30 NCE) of a) the protonated aPB derivative of standard PI (18:1 Δ9/18:1 Δ9); b) the protonated aPB derivative of PE (18:1/18:1); c) the protonatedaPB derivative of PC (18:1/18:1). The diagnostic ions are marked with a red circle.

Table S1. Detailed LipidSearch parameters for polar lipids data processing.

**Instrument**	HCD
**Peak Detection**	
Recalc isotope	on
R.T. interval (min)	0.01
**Search Options**	
ExpType	LC-MS
Parent tol.	0.1 Da
NL/Prec tol.	0.5 Da
Precursor tol.	5.0 ppm
Product tol.	8.0 ppm
Merge Range (min.)	2.0
Min Peak Width (min.)	0.0
Intensity threshold	0.01 parent ion, threshold type: relative 1.0 product ion
m-Score threshold	2.0
**Compound Rt Correction**	
m/z tolerance	0.005 amu + 0.00 ppm
Warping based RT correction	Activated
Within batch Rt tolerance	0.1 min
Between batch Rt tolerance	0.2 min
Rt window	1 min
**Target class**	
Phospholipids	Lysophosphatidic acid, phosphatidic acid, lysophosphatidylcholine, phosphatidylcholine, lysophosphatidylethanolamine, phosphatidylethanolamine, lysophopshatidylglycerol, phosphatidylglycerol, lysophosphatidylinositol, phosphatidylinositol, lysophosphatidylserine, phosphatidylserine
Sphingolipids	Ceramides, glucosylsphingosine, lysosphingomyelin, sphingomyelin
**Adducts**	
negative	-H, +HCOO, +CH_3COO, -2H, -CH_3

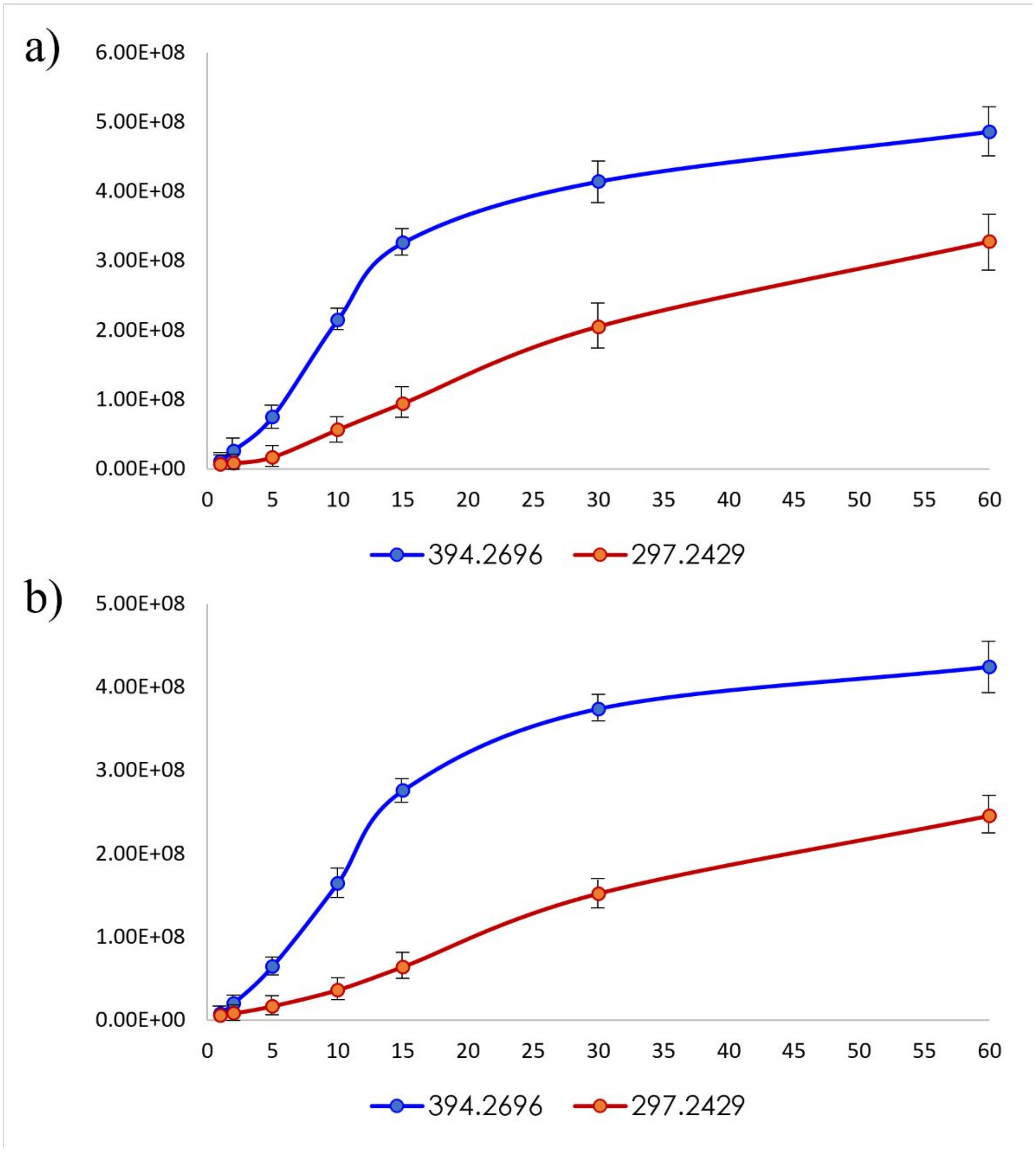

Figure S1. Dispersion graphs for FA 18:1 Δ9 (a) and FA 18:1 Δ11 (b) showing the total peak areas of *m/z* 394.2696 (6-AU derivative) and *m/z* 297.2429 (oxidized FA 18:1).

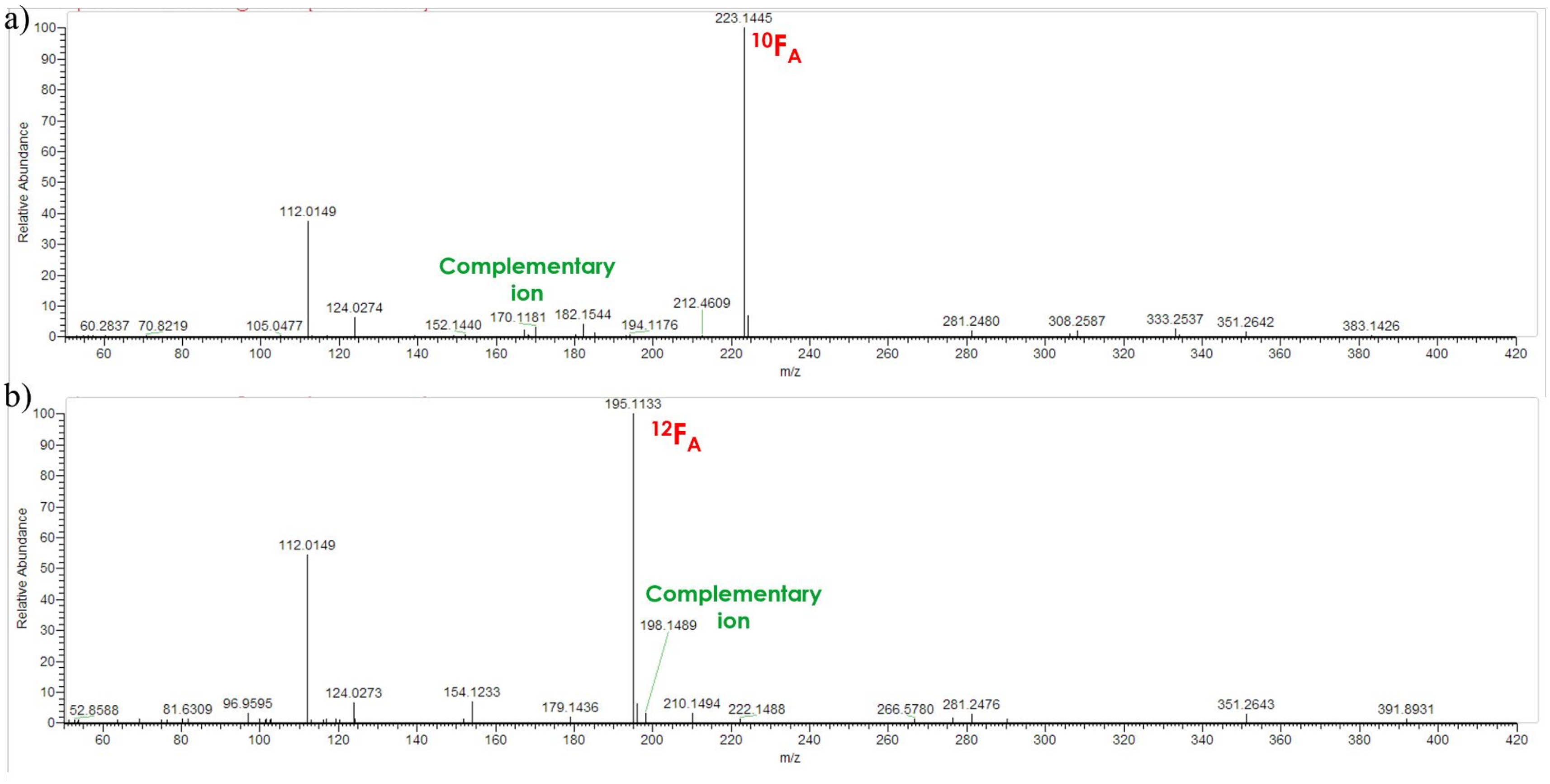

Figure S2. MS/MS scan associated to the aPB derivative P_A of FA 18:1 Δ9 (a) and FA 18:1 Δ11 (b). Other than the main F_A ion (base peak), the complementary ion is visible in the spectrum (m/z 170.1181 for 18:1 Δ9 and m/z 198.1489 for 18:1 Δ11)

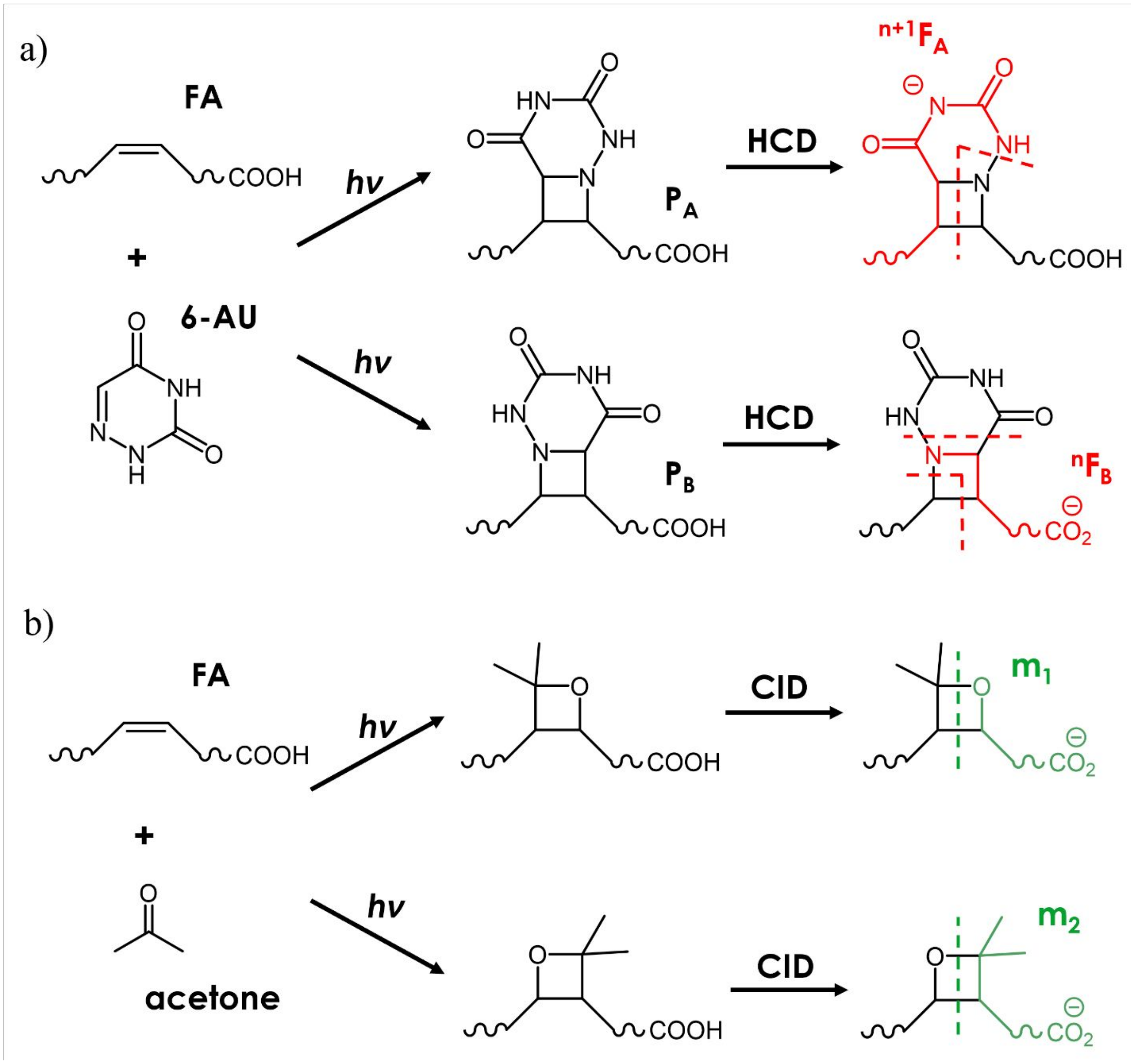

Figure S3. Comparison of the fragmentation pathways that lead to the diagnostic product ions between aPB reaction with 6-AU (a) and PB reaction with acetone (b).

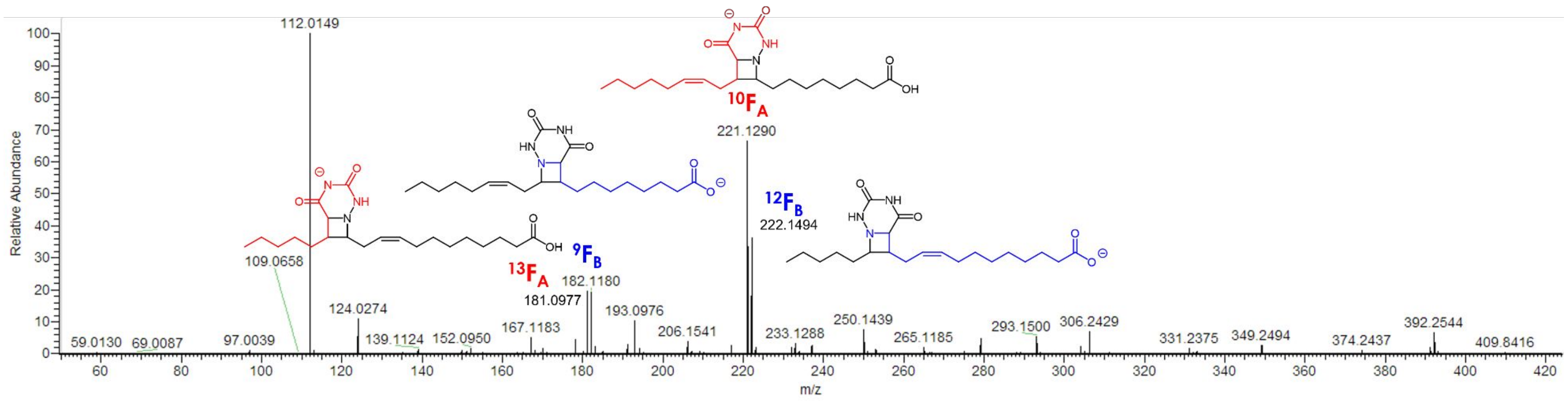

Figure S4. ESI(−)-HCD MS/MS spectrum (40 NCE) of deprotonated FA 18:2 Δ9,12 after photochemical reaction with 6-AU.

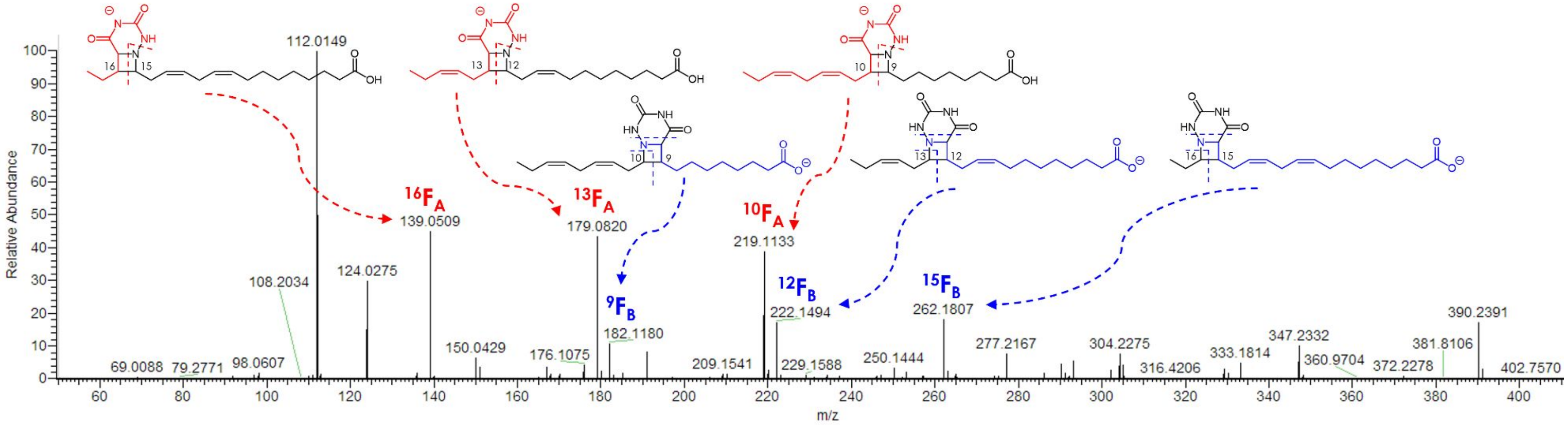

Figure S5. ESI(−)-HCD MS/MS spectrum (40 NCE) of deprotonated FA 18:3 Δ9,12,15 after photochemical reaction with 6-AU.

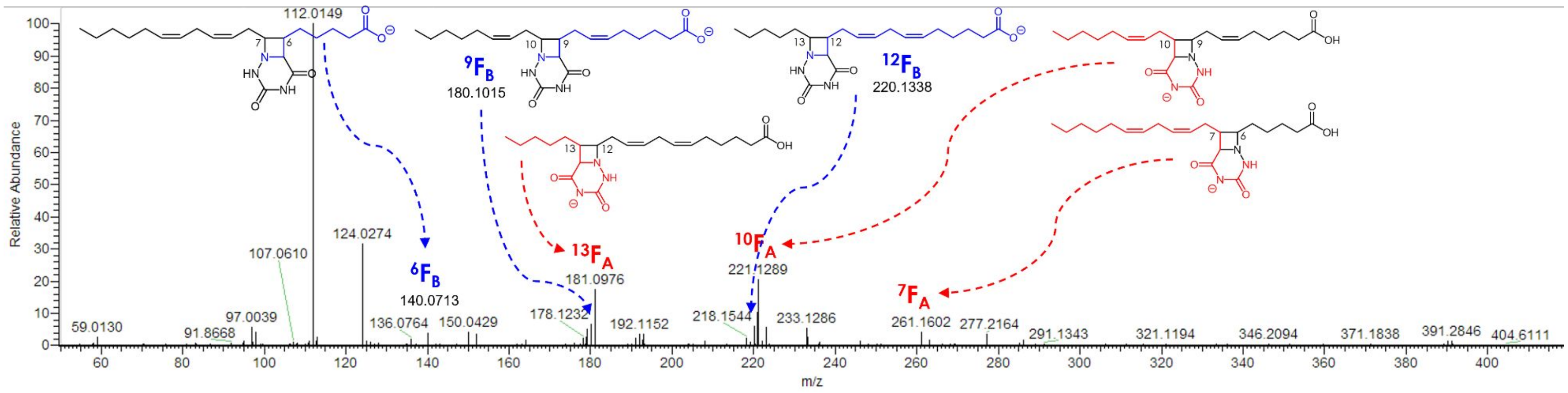

Figure S6. ESI(−)-HCD MS/MS spectrum (40 NCE) of deprotonated FA 18:3 Δ6,9,12 after photochemical reaction with 6-AU.

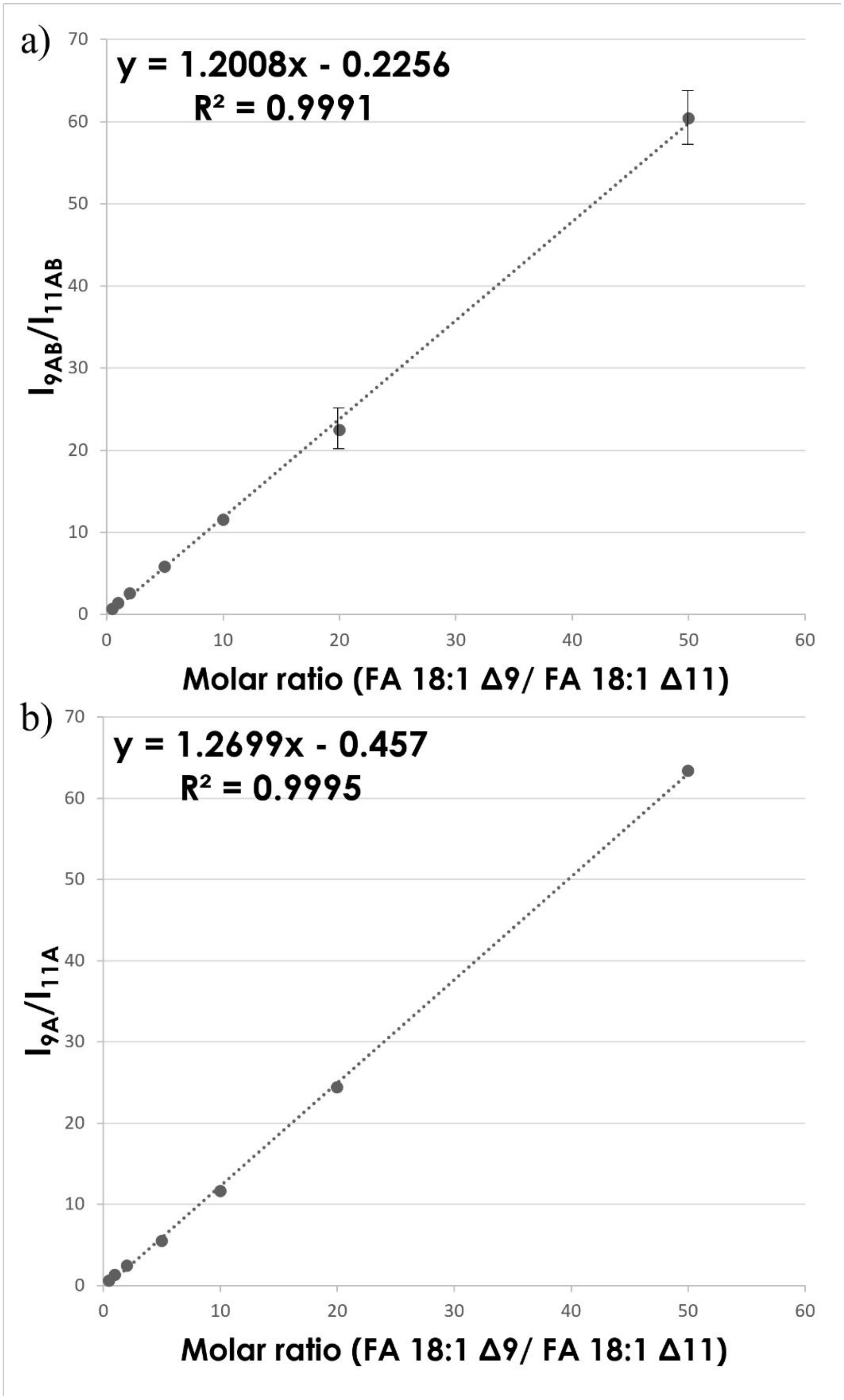

Figure S7. Linear relationship between the molar ratios of FA 18:1 Δ9 and Δ11 and: (a) the intensities of the diagnostic ion pairs (I_{9AB}/I_{11AB}), (b) the intensities of the diagnostic F_A ions (I_{9A}/I_{11A}).

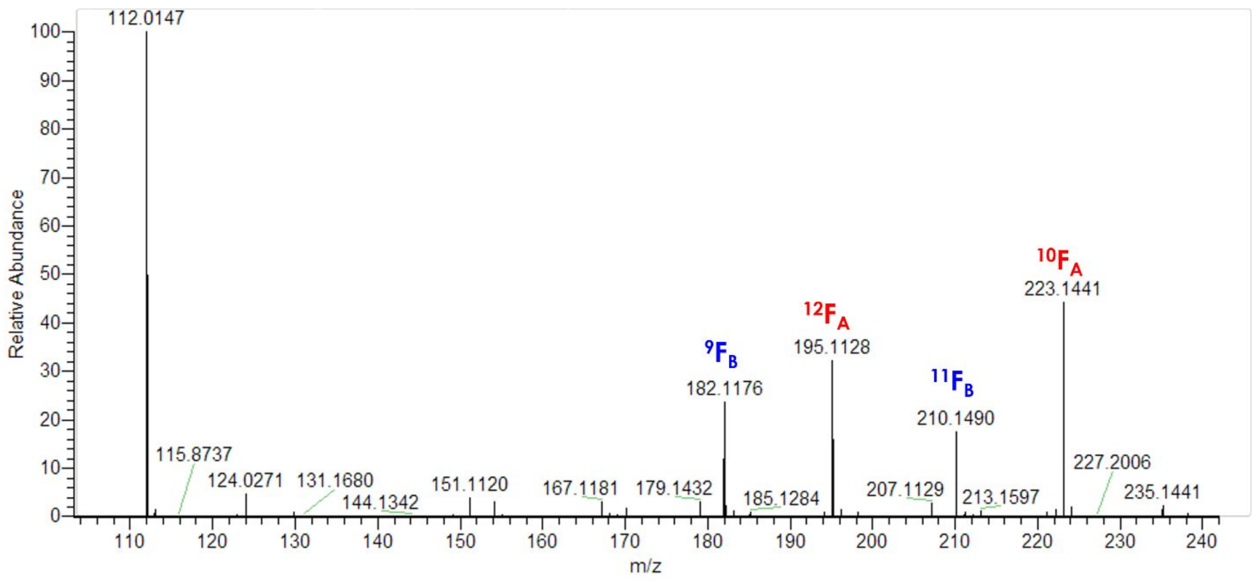

Figure S8. ESI(−)-HCD MS/MS spectrum (40 NCE) of FA 18:1 Δ9 and Δ11 at equal molar ratio after aPB derivatization.

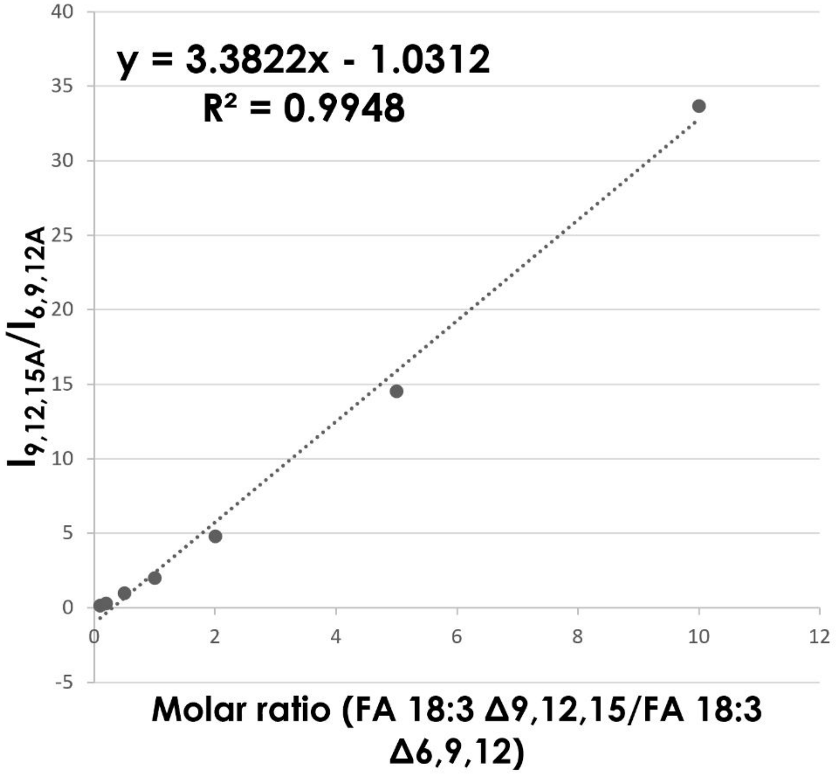

Figure S9. Linear relationship between the molar ratios of FA 18:3 Δ9,12,15 and Δ6,9,12 and the intensities of the diagnostic F_A ions ($I_{9,12,15A}/I_{6,9,12A}$).

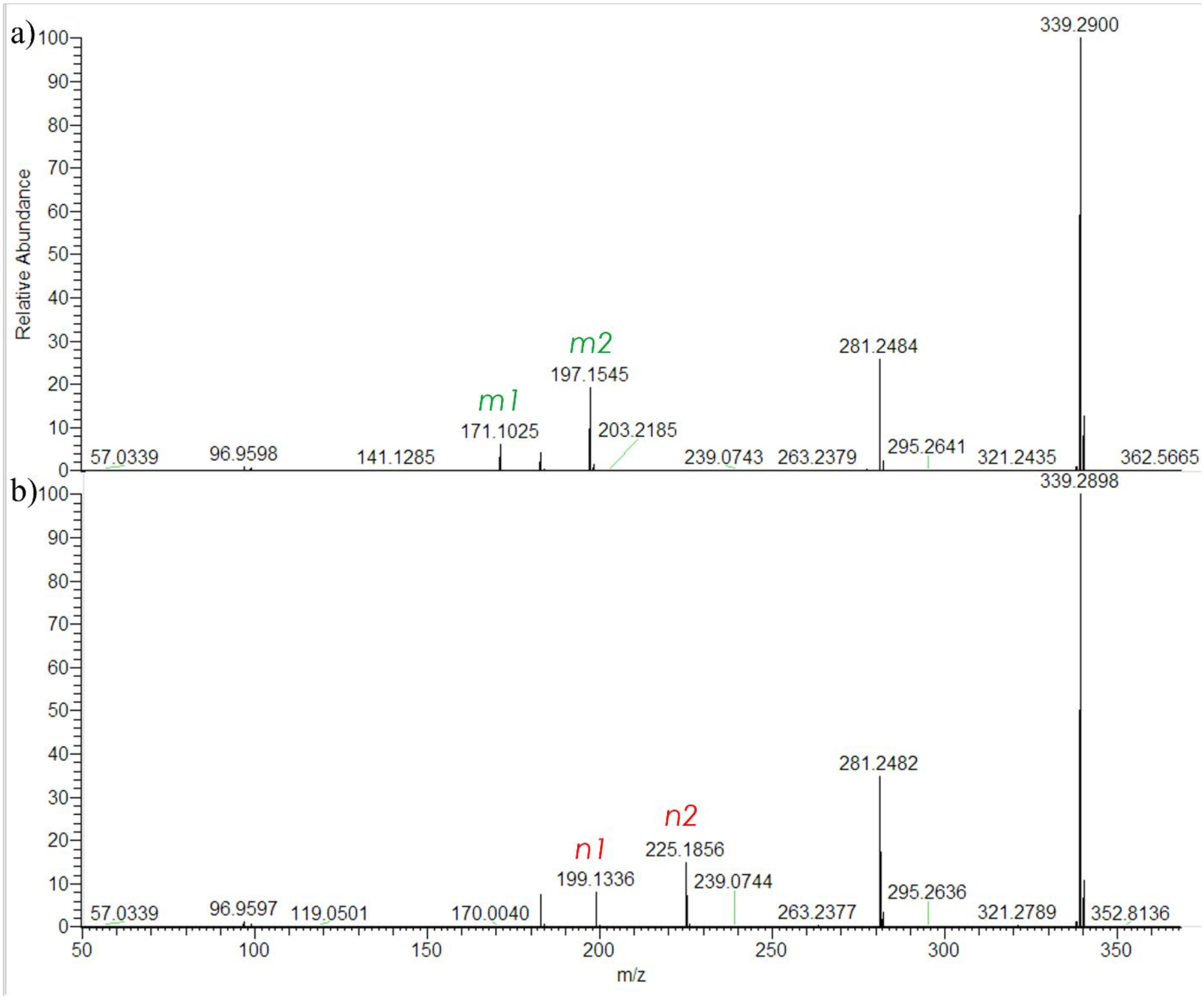

Figure S10. ESI(−)-HCD MS/MS spectrum (40 NCE) of (a) deprotonated FA 18:1 Δ9 and (b) deprotonated FA 18:1 Δ11 after PB derivatization with acetone. The reported nomenclature of the product ions is that of Ma (https://doi.org/10.1073/pnas.1523356113), with the two ions deriving from each of the two main PB products.

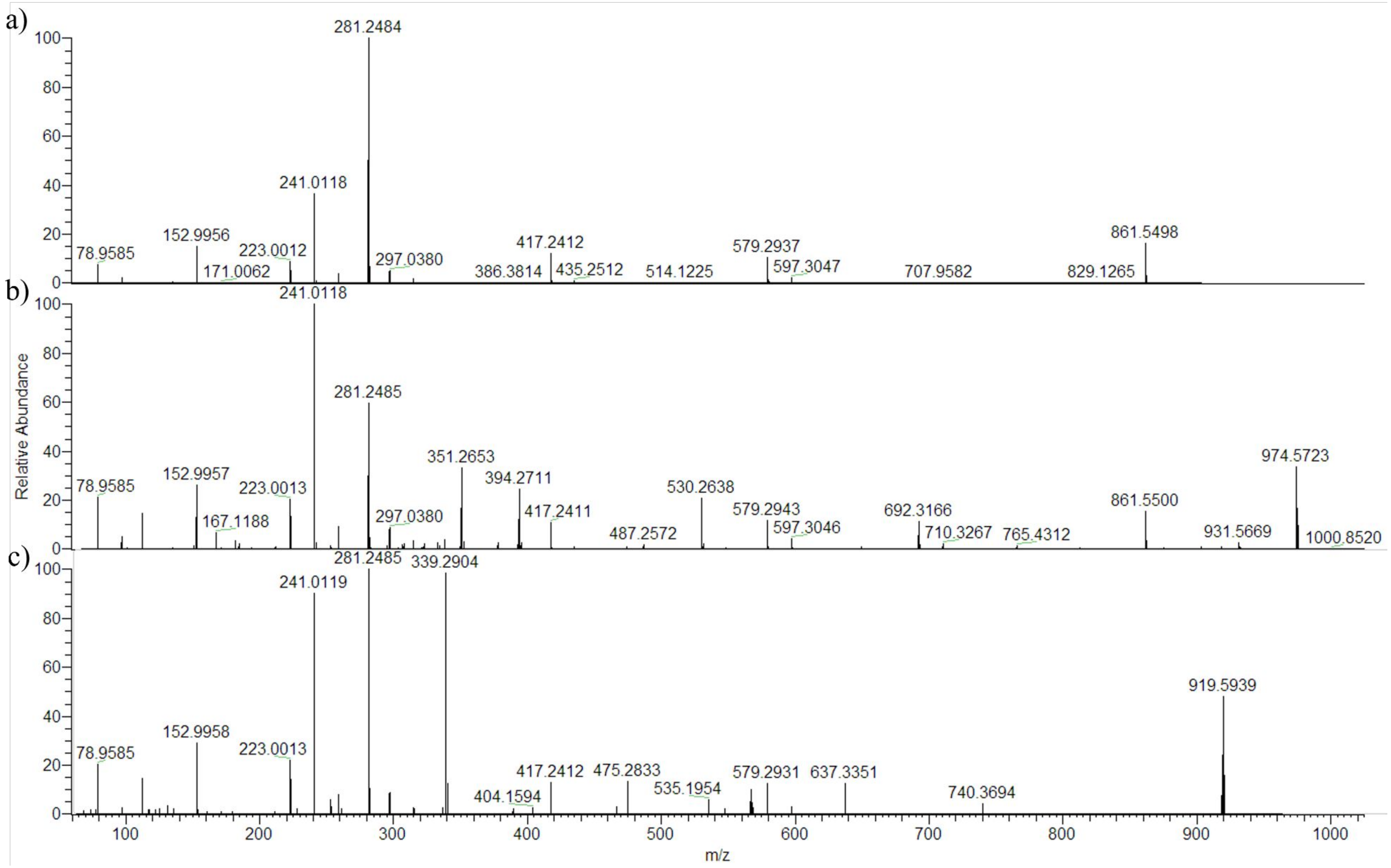

Figure S11. ESI(–)-HCD MS/MS spectrum (30 NCE) of a) underivatized PI (18:1/18:1); b) aPB derivative of PI (18:1/18:1); c) PB derivative of PI (18:1/18:1).

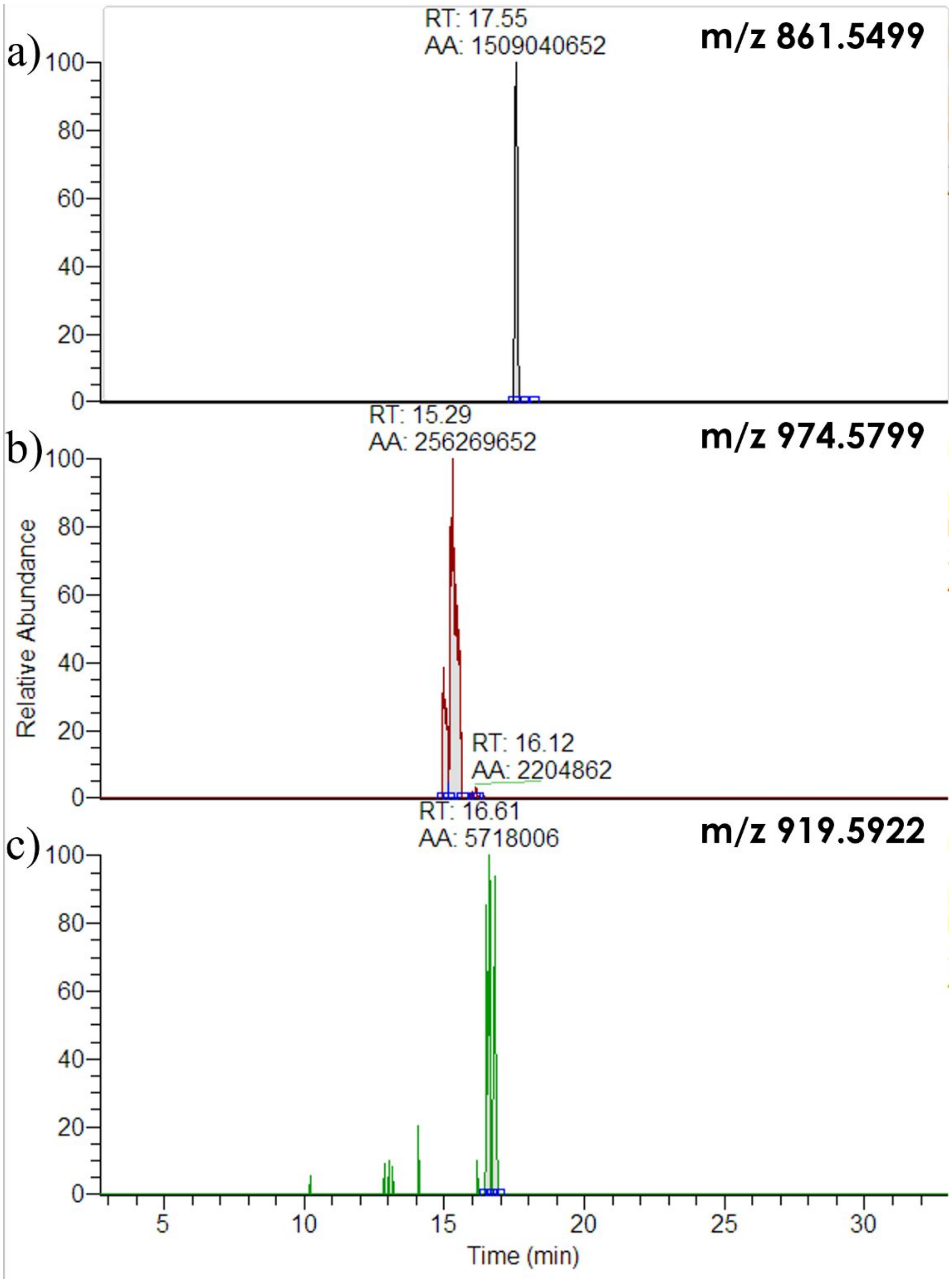

Figure S12. Total ion current of a) deprotonated underivatized PI (18:1/18:1); b) deprotonated aPB derivative of PI (18:1/18:1); c) deprotonated PB derivative of PI (18:1/18:1) obtained by the competitive aPB/PB reaction.

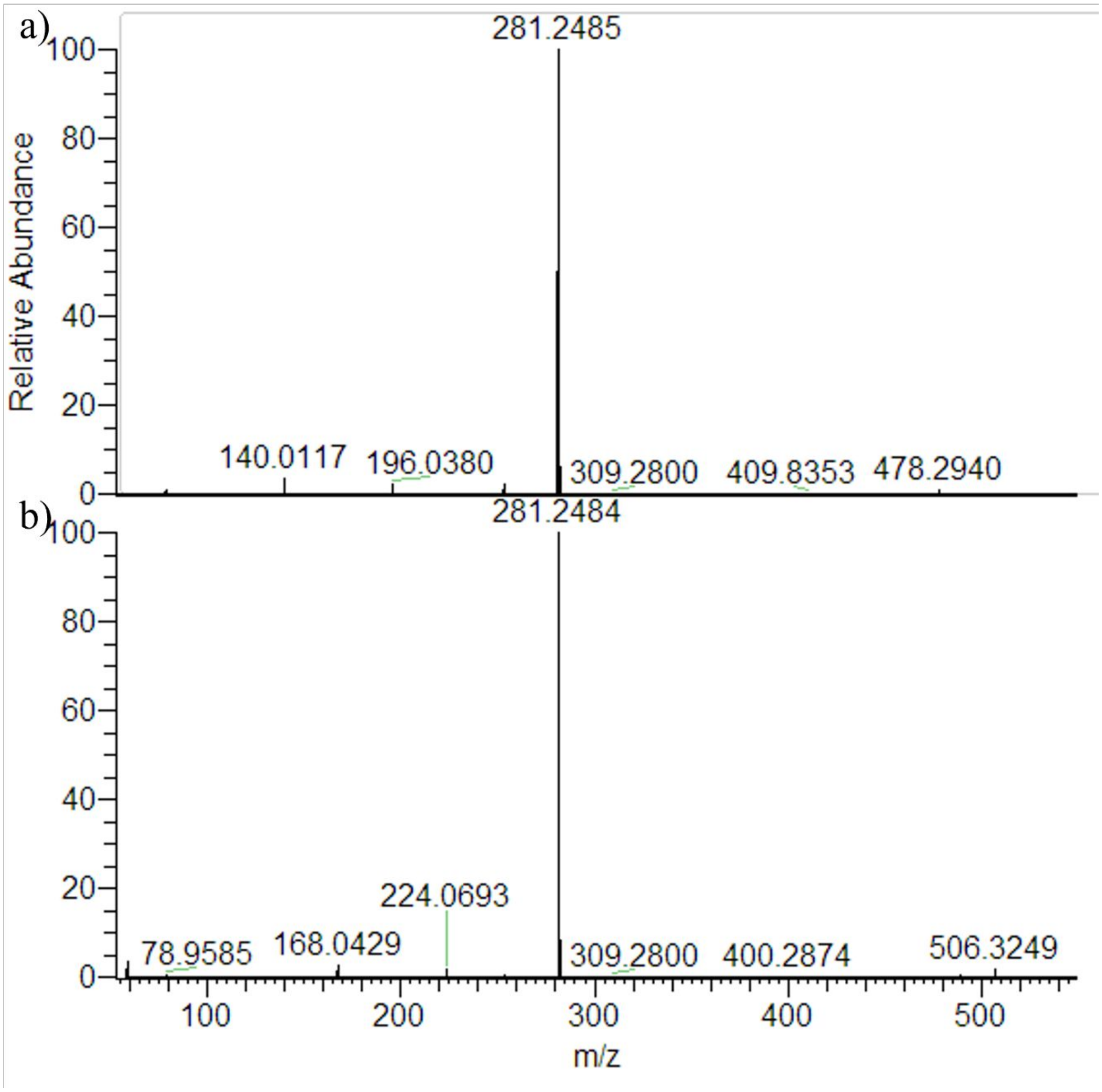

Figure S13. ESI(–)-HCD MS/MS spectrum (30 NCE) in negative ion mode of a) underivatized deprotonated PE (18:1/18:1); b) underivatized acetate adduct of PC (18:1/18:1).

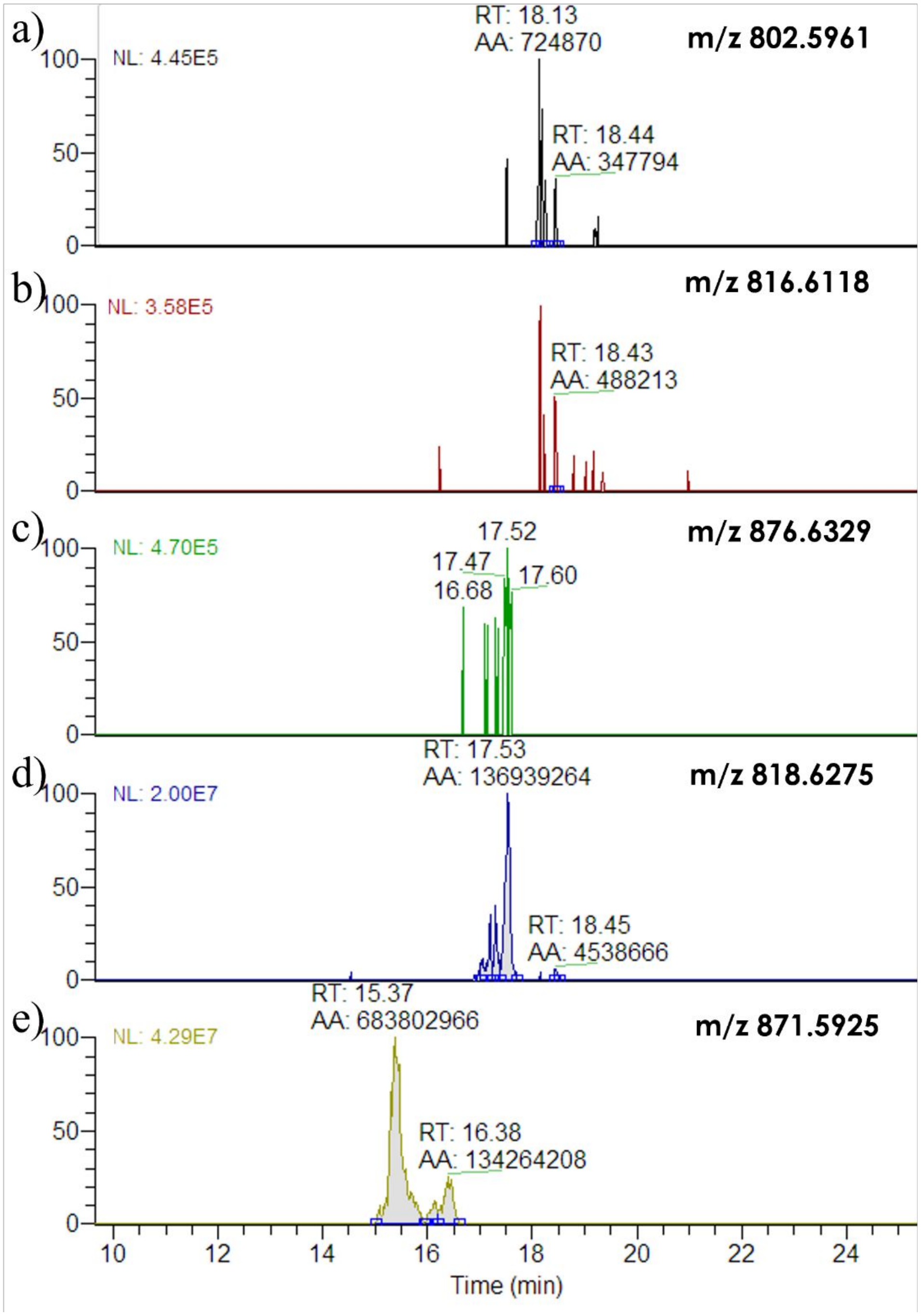

Figure S14. Total ion current of the m/z corresponding to a) the deprotonated PB derivative of PC (16:0/18:1); b) the demethylated PB derivative of PC (16:0/18:1); c) acetate adduct of the PB derivative of PC (16:0/18:1); d) the protonated PB derivative of PC (16:0/18:1); e) the deprotonated aPB derivative of PC (16:0/18:1).

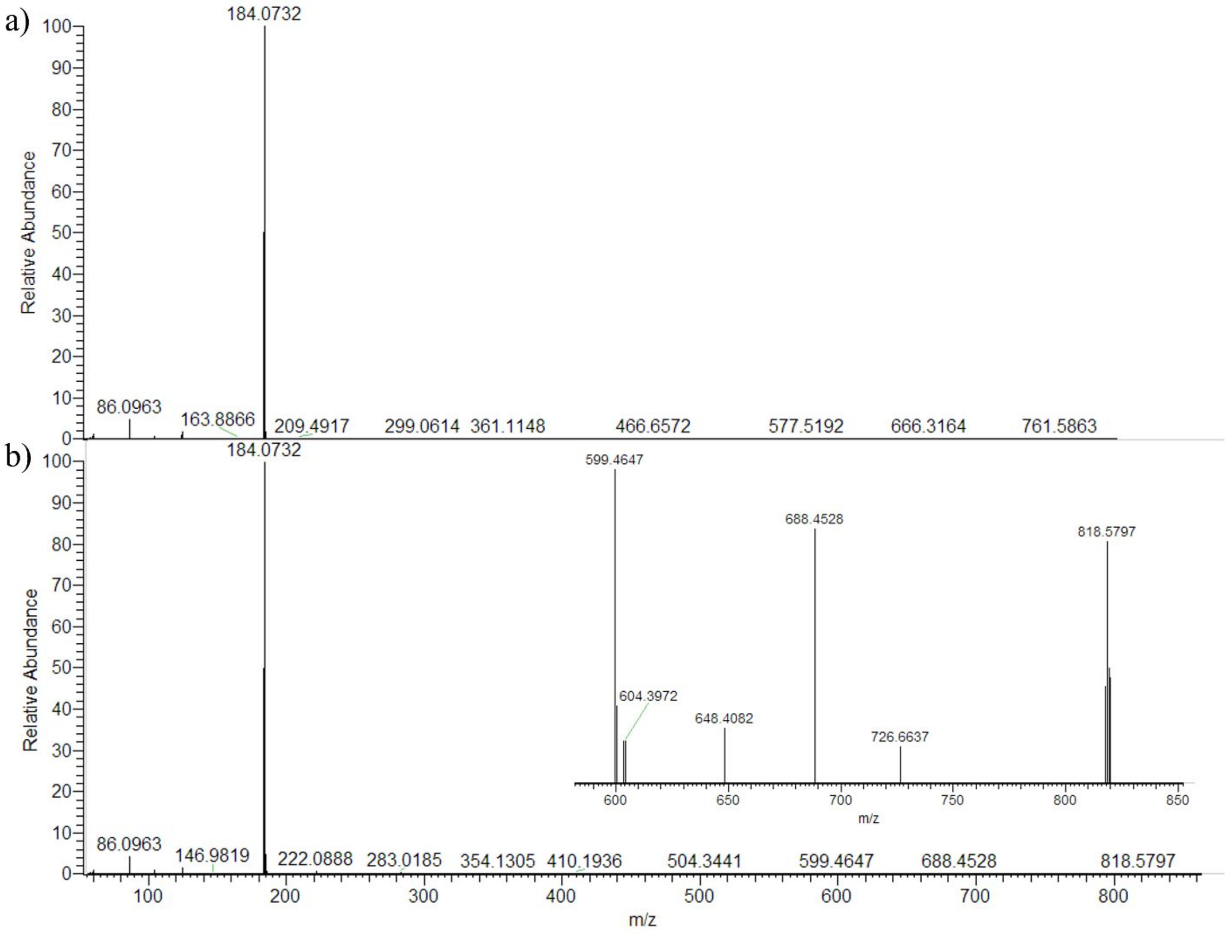

Figure S15. ESI(+)-HCD MS/MS spectrum (30 NCE) of (a) protonated underivatized PC (16:0/18:1) and (b) protonated PB-derivatized PC (16:0/18:1).

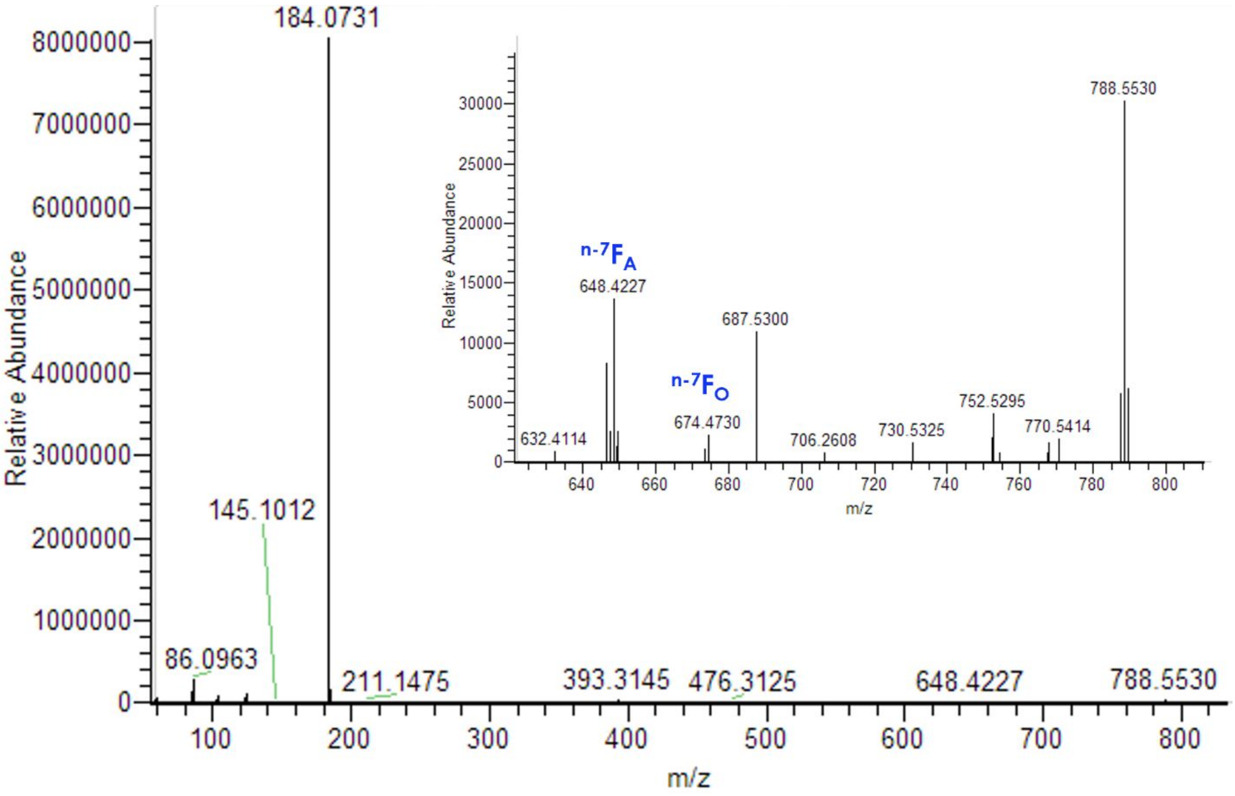

Figure S16. ESI(+)-HCD MS/MS spectrum (30 NCE) of protonated PB-derivatized PC (16:1/16:1) from the yeast extract. A zoom on the high m/z range shows a low abundance of the typical diagnostic peaks for pinpointing carbon-carbon double bonds.

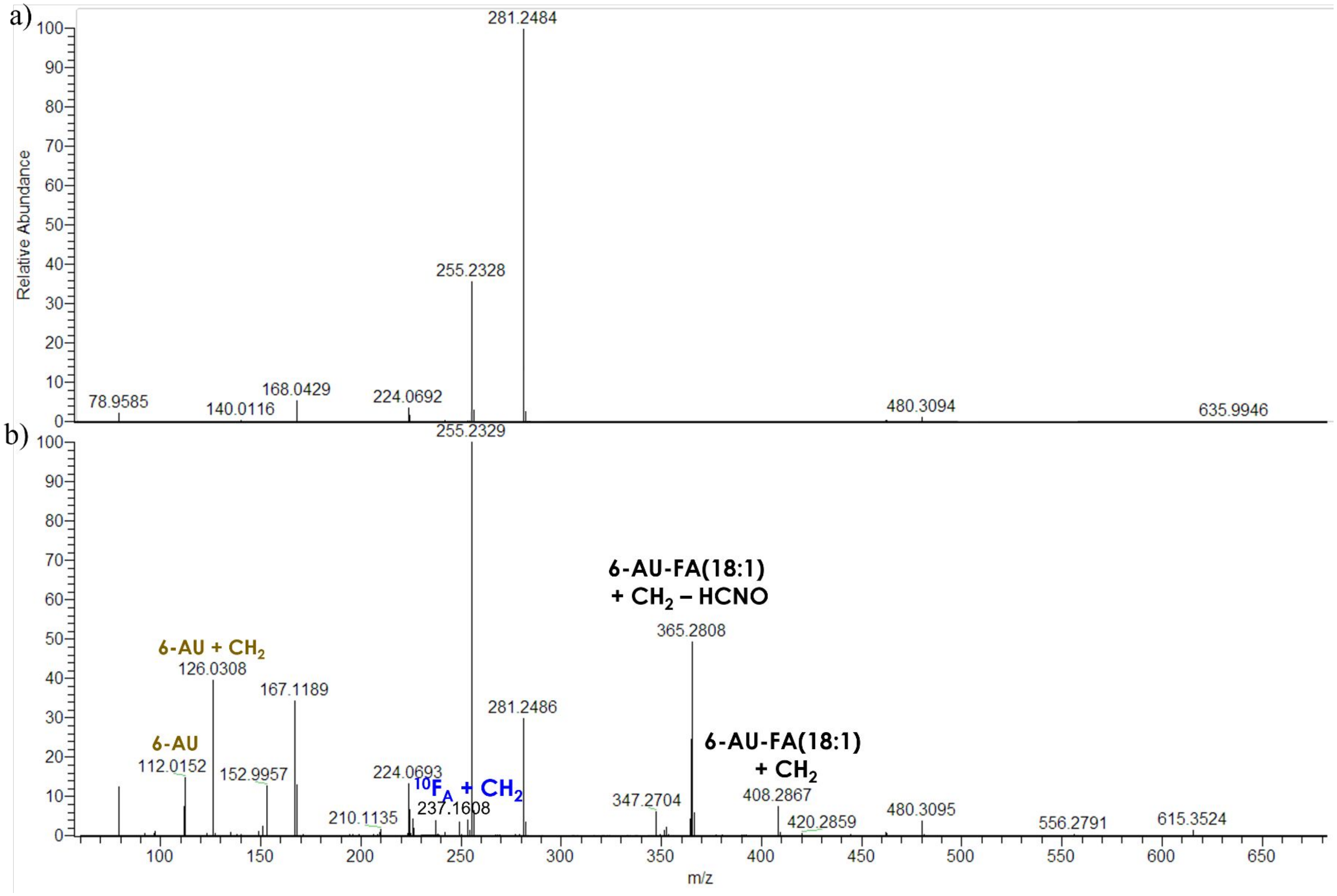

Figure S17. ESI(−)-HCD MS/MS spectrum (30 NCE) in negative ion mode of a) underivatized acetate adduct of standard PC (16:0/18:1); b) deprotonated aPB derivative of standard PC (16:0/18:1).

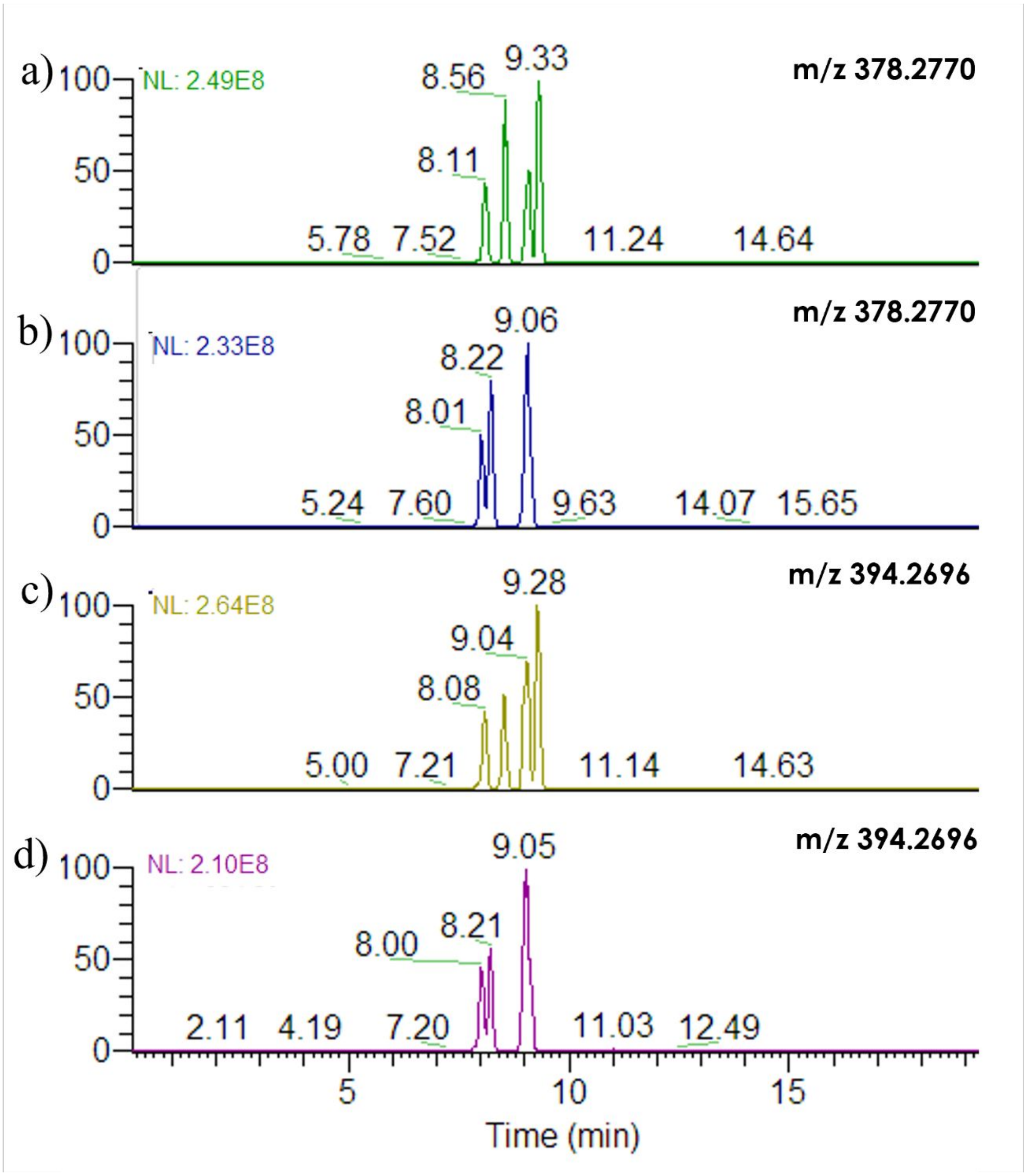

Figure S18. Total ion current of the m/z corresponding to a) the protonated in-source fragment of the aPB derivative of FA 18:1 Δ9; b) the protonated in-source fragment of the aPB derivative of FA 18:1 Δ11; c) the deprotonated aPB derivative of FA 18:1 Δ9; d) the deprotonated aPB derivative of FA 18:1 Δ11.

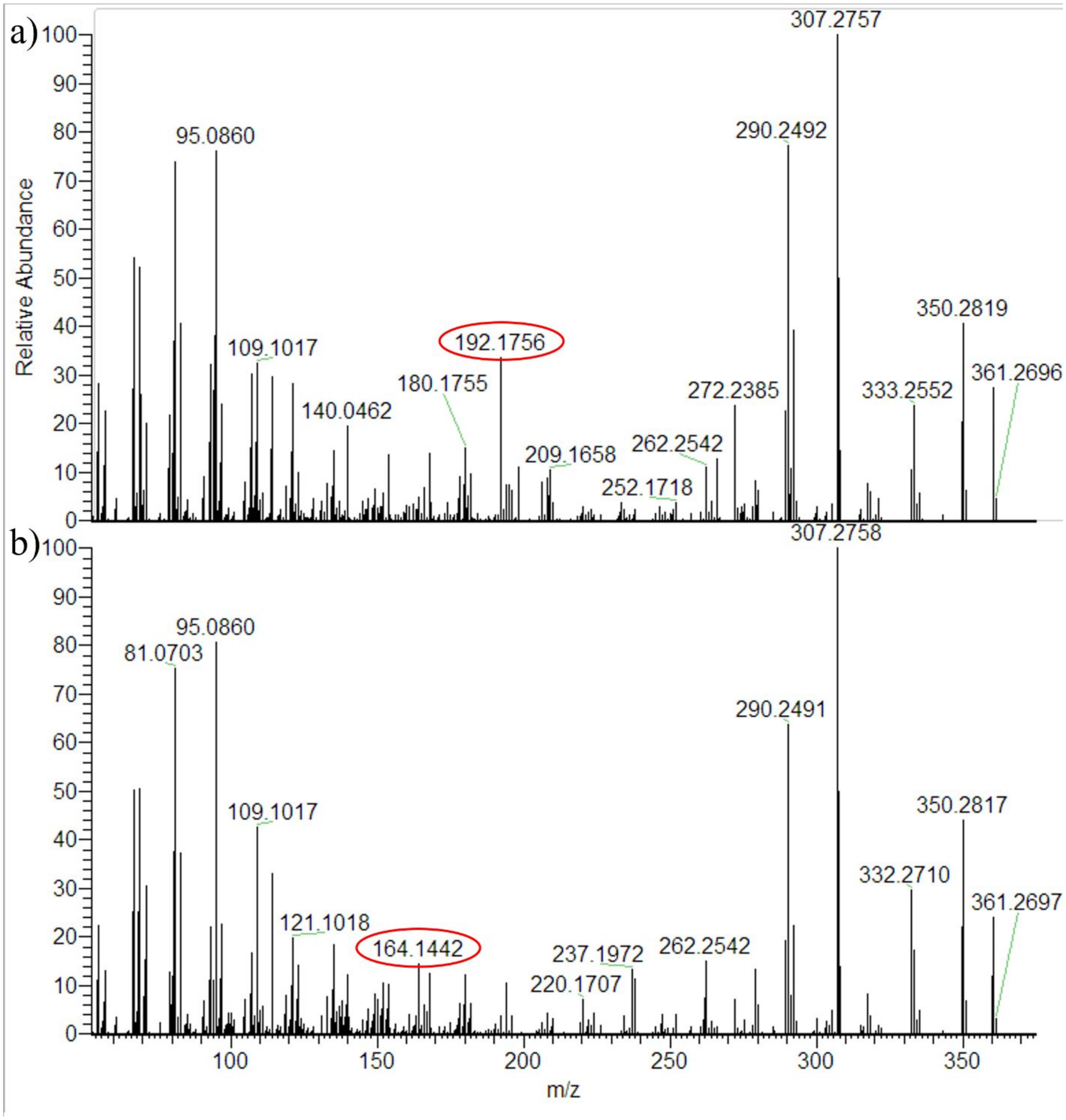

Figure S19. ESI(+)-HCD MS/MS spectrum (30 NCE) of a) the protonated aPB derivative of FA 18:1 Δ9; b) the protonated aPB derivative of FA 18:1 Δ11. The diagnostic ions are marked with a red circle. The relatively high HCD energy employed could explain the obtention of several ions from secondary processes in the low m/z range.

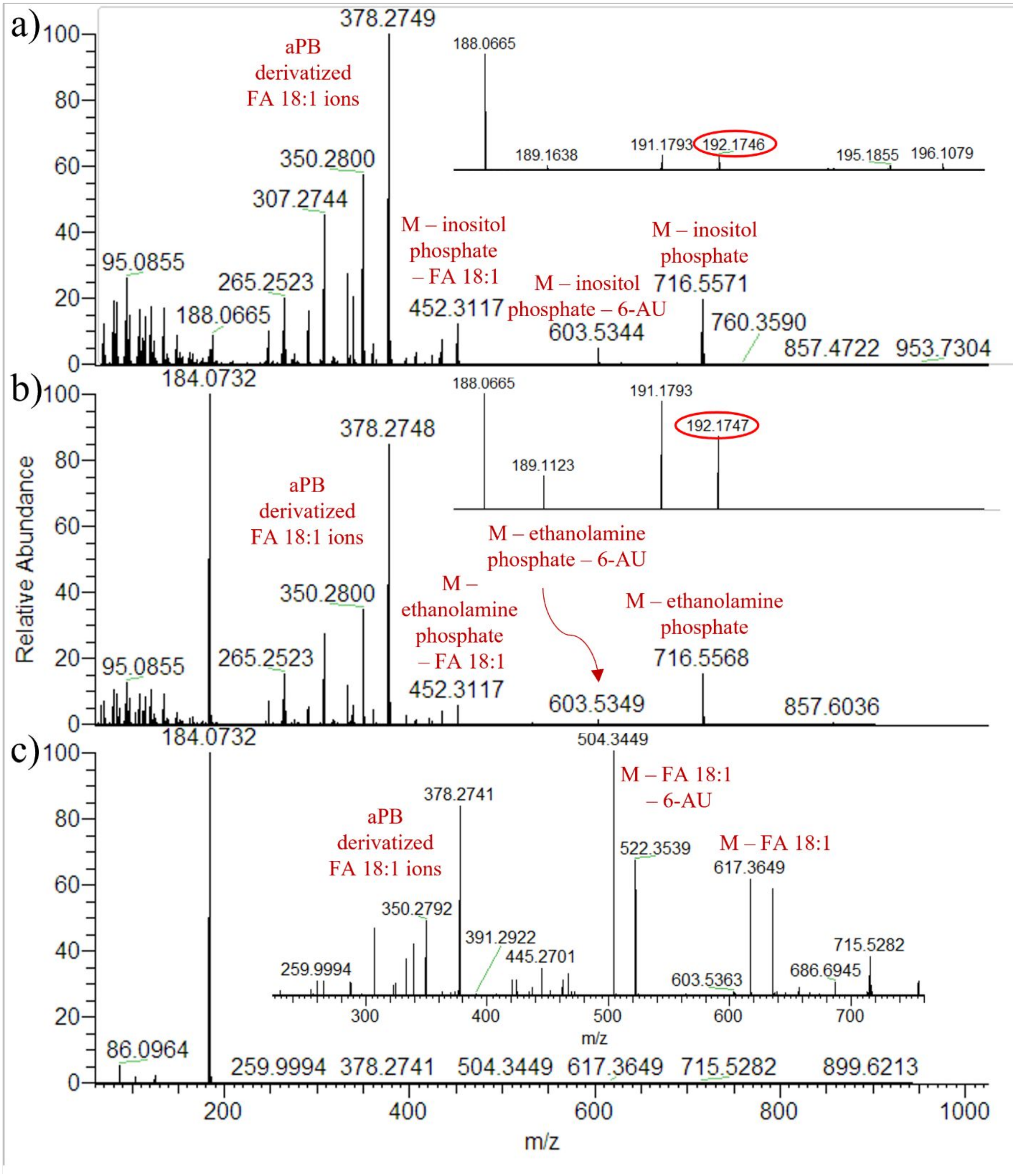

Figure S20. ESI(+)-HCD MS/MS spectrum (30 NCE) of a) the protonated aPB derivative of standard PI (18:1 Δ9/18:1 Δ9); b) the protonated aPB derivative of PE (18:1/18:1); c) the protonated aPB derivative of PC (18:1/18:1). The diagnostic ions are marked with a red circle. The

relatively high HCD energy employed could explain the obtention of several ions from secondary processes in the low m/z range.
